# Cholera epidemic in Yemen, 2016–18: an analysis of surveillance data

**DOI:** 10.1016/S2214-109X(18)30230-4

**Published:** 2018-05-03

**Authors:** Anton Camacho, Malika Bouhenia, Reema Alyusfi, Abdulhakeem Alkohlani, Munna Abdulla Mohammed Naji, Xavier de Radiguès, Abdinasir M Abubakar, Abdulkareem Almoalmi, Caroline Seguin, Maria Jose Sagrado, Marc Poncin, Melissa McRae, Mohammed Musoke, Ankur Rakesh, Klaudia Porten, Christopher Haskew, Katherine E Atkins, Rosalind M Eggo, Andrew S Azman, Marije Broekhuijsen, Mehmet Akif Saatcioglu, Lorenzo Pezzoli, Marie-Laure Quilici, Abdul Rahman Al-Mesbahy, Nevio Zagaria, Francisco J Luquero

**Affiliations:** a Epicentre, Paris, France; b London School of Hygiene & Tropical Medicine, London, UK; c World Health Organization, Sana'a, Yemen; d Health Authorities, Yemen; e Central Public Health Laboratory, Sana'a, Yemen; f World Health Organization, Cairo, Egypt; g Médecins Sans Frontières, Dubai, United Arab Emirates; h Médecins Sans Frontières, Barcelona, Spain; i Médecins Sans Frontières, Geneva, Switzerland; j Médecins Sans Frontières, Amsterdam, Netherlands; k Médecins Sans Frontières, Sana'a, Yemen; l World Health Organization, Geneva, Switzerland; m Johns Hopkins School of Public Health, Baltimore, MD, USA; n UNICEF, Sana'a, Yemen; o National Reference Center for Vibrios and Cholera, Institut Pasteur, Paris, France

## Abstract

**Background:**

In war-torn Yemen, reports of confirmed cholera started in late September, 2016. The disease continues to plague Yemen today in what has become the largest documented cholera epidemic of modern times. We aimed to describe the key epidemiological features of this epidemic, including the drivers of cholera transmission during the outbreak.

**Methods:**

The Yemen Health Authorities set up a national cholera surveillance system to collect information on suspected cholera cases presenting at health facilities. Individual variables included symptom onset date, age, severity of dehydration, and rapid diagnostic test result. Suspected cholera cases were confirmed by culture, and a subset of samples had additional phenotypic and genotypic analysis. We first conducted descriptive analyses at national and governorate levels. We divided the epidemic into three time periods: the first wave (Sept 28, 2016, to April 23, 2017), the increasing phase of the second wave (April 24, 2017, to July 2, 2017), and the decreasing phase of the second wave (July 3, 2017, to March 12, 2018). We reconstructed the changes in cholera transmission over time by estimating the instantaneous reproduction number, *R*_t_. Finally, we estimated the association between rainfall and the daily cholera incidence during the increasing phase of the second epidemic wave by fitting a spatiotemporal regression model.

**Findings:**

From Sept 28, 2016, to March 12, 2018, 1 103 683 suspected cholera cases (attack rate 3·69%) and 2385 deaths (case fatality risk 0·22%) were reported countrywide. The epidemic consisted of two distinct waves with a surge in transmission in May, 2017, corresponding to a median *R*_t_ of more than 2 in 13 of 23 governorates. Microbiological analyses suggested that the same *Vibrio cholerae* O1 Ogawa strain circulated in both waves. We found a positive, non-linear, association between weekly rainfall and suspected cholera incidence in the following 10 days; the relative risk of cholera after a weekly rainfall of 25 mm was 1·42 (95% CI 1·31–1·55) compared with a week without rain.

**Interpretation:**

Our analysis suggests that the small first cholera epidemic wave seeded cholera across Yemen during the dry season. When the rains returned in April, 2017, they triggered widespread cholera transmission that led to the large second wave. These results suggest that cholera could resurge during the ongoing 2018 rainy season if transmission remains active. Therefore, health authorities and partners should immediately enhance current control efforts to mitigate the risk of a new cholera epidemic wave in Yemen.

**Funding:**

Health Authorities of Yemen, WHO, and Médecins Sans Frontières.

## Introduction

Yemenis continue to endure the devastating consequences of the war that erupted in March, 2015. While this conflict has officially caused at least 8757 deaths among civilians and injured more than 50 000 people, its broader impact has severely disrupted society and infrastructure.^1^ 3 million people have been displaced and the health system has lost capacity to provide even basic services, with 55% of health facilities being no longer fully functional.^2^ Moreover, with damaged water supply infrastructure, chronic water scarcity, and surging water prices, the UN Office for the Coordination of Humanitarian Affairs estimates that more than 50% of the 29·9 million Yemenis are in need of water and sanitation assistance.^3^, ^4^ Against the backdrop of this crisis, the largest reported cholera epidemic to date is increasing the suffering of this vulnerable population.

Even healthy individuals can die of cholera within hours after symptoms begin.^5^ *Vibrio cholerae* produces a profuse watery diarrhoea that can quickly progress to dehydration and hypovolaemic shock, and can kill up to 50% of patients if fluids are not properly replaced.^6^ However, the case fatality risk (CFR) can be less than 1% with appropriate case management.^5^ Scarcity of adequate treatment is more common during the initial phase of unexpected outbreaks and in crisis settings. Inadequate preparedness has determined much of the mortality burden associated with cholera during some of the largest contemporary epidemics.^7^, ^8^, ^9^

**Research in context**

**Evidence before this study**

We did a PubMed search with dates between Jan 1, 1970, and March 14, 2018, for studies published in English using the search terms “cholera” [title] AND (outbreak OR rain OR (infection AND immunity)). We then manually selected relevant articles on the basis of the title and by reading the abstracts. Additionally, we obtained the history of cholera outbreaks in Yemen since 1971 from the Global Health Observatory data repository. The seventh cholera pandemic has caused a large burden of disease worldwide for more than 50 years, with the highest burden of disease in Africa, the Americas, and southern Asia. Explosive cholera outbreaks have been observed in the past 10 years years in several African countries and Haiti. Some of these outbreaks have been observed during rainy seasons and some studies have found positive associations between rainfall and cholera risk.

Seventh pandemic strains have spread in the Middle East causing outbreaks in several countries, including Yemen, although without reaching the size of the outbreaks reported in Africa or Haiti. Yemen reported a limited number of cholera cases to WHO in ten out of 40 years from 1971 to 2010, with the largest number (1953 suspected cholera cases) reported in 1979. In 2011, a larger cholera outbreak was reported with 31 789 cases and 45 deaths. After a lull of 5 years, a new cholera outbreak started in 2016 and continued to spread in 2017, becoming the largest documented cholera epidemic of modern times with more than 1 million suspected cholera cases reported so far. By contrast with previous nationwide cholera outbreaks such as in Haiti, the outbreak in Yemen featured a small first wave in 2016 followed by a larger second wave in 2017.

**Added value of this study**

Understanding what factors drove the cholera epidemic in Yemen is key to the design of tailored public-health interventions and assessment of the risk and location of future waves. To our knowledge, our study is the first to quantify the size, spatial extent, and key populations affected by the two epidemic waves in Yemen and determine the plausible drivers of cholera transmission during the course of the outbreak. We found that the small first cholera epidemic wave in 2016 had a key role in seeding the bacteria across Yemen during the dry season. When the rains returned in April, 2017, they triggered widespread cholera transmission that led to the large second wave observed during the following months. This conclusion is further supported by our microbiological analyses, which suggest that the resurgence of the epidemic in 2017 is unlikely to have resulted from the introduction of a new *Vibrio cholerae* strain.

**Implications of all the available evidence**

Our findings have important operational implications for Yemen and for other countries facing cholera epidemics. In Yemen, with the return of the 2018 rainy season, a potential third wave could occur, which would further weaken a very vulnerable population. This report thus calls for urgent action to enhance current control efforts, including the epidemiological and microbiological surveillance, vaccination, and water and sanitation interventions, all which need a large commitment from local officials, donors, and international partners to stop the spread of cholera.

The largest previous outbreaks of the current seventh cholera pandemic, including those from Haiti,^7^ Zimbabwe,^10^ and Goma (Democratic Republic of the Congo),^11^ share two common characteristics: an initial explosion of cases and difficulty providing an adequate medical response. Together, these translated into a massive surge of cases and a relatively high fatality risk during the first weeks of the epidemics. By contrast, the outbreak in Yemen, which began in late September, 2016, featured a small first wave, a larger second wave (beginning in late April, 2017), and a low CFR, despite the highly precarious situation in the country. As such, the factors that drove the cholera epidemic in Yemen are unclear. This uncertainty hampers design of tailored public-health interventions and assessment of risk and location of future waves.

In this study, we quantified the size, spatial extent, and key populations affected by the two epidemic waves in Yemen and attempted to determine the plausible drivers of cholera transmission during the course of the outbreak.

## Methods

### Cholera surveillance system

The Yemen Health Authorities with support from WHO set up an electronic integrated disease early warning system in 2013, which gradually increased from 98 reporting sites to 1982 by the end of 2016.^12^ Upon confirmation of the first cholera case, public and private health facilities that were part of this countrywide cholera surveillance system collated suspected cases using a common line-list database (Excel 2010, Microsoft). Following the recommendations of the surveillance working group of the Global Task Force on Cholera Control,^13^ a suspected case was any patient presenting with three or more liquid stools with or without vomiting in the past 24 h. A confirmed case was a suspected case with *V cholerae* O1 or O139 confirmed by culture. This database contains dates of disease onset and admission, age, sex, district of origin, dehydration severity (based on the WHO severity grading^5^), laboratory results when available, and disease outcome (discharge or death). District surveillance officers (for each of the 333 districts) compiled the line-list from all health facilities in their district. This district line-list database was sent electronically to the governorate level (23 total governorates) each day. Data were aggregated by the Emergency Operation Centre run by the Yemen Health Authorities and cleaned by WHO surveillance officers to remove duplicates, standardise district names, and solve inconsistent entries (eg, inverted month and day in date format).

### Laboratory analysis

Governorates provided periodic samples of stool specimens from suspected cases to confirm the presence of *V cholerae* using standard culture methods.^14^ In addition, ten *V cholerae* isolates from the 2016 epidemic wave and 31 from 2017 were selected by convenience sampling and sent to the French National Reference Center for Vibrios and Cholera (Pasteur Institute, Paris, France) for additional phenotypic and genotypic analysis. Cholera rapid diagnostic tests (Crystal VC, Span Diagnostics, Surat, India) were supplied by the Yemen Health Authorities with the recommendation to perform a rapid diagnostic test on one in ten suspected cases in each cholera treatment centre.

### Rainfall data

Because of the scarcity of functional ground weather stations in Yemen since the start of the war (appendix p 13), rainfall data were estimated indirectly from satellite imagery. High resolution (0·05°) gridded daily precipitation data were obtained for 2016–18 from the Climate Hazards Group Infrared Precipitation with Stations (CHIRPS) data.^15^ Daily precipitation time series for each district were computed by a weighted mean of the grid cells, with weights proportional to the fraction of each grid cell contained within the district.

### Data analysis

We calculated weekly time series of suspected cholera cases, CFR (ratio of the numbers of suspected deaths over suspected cases), proportion of cases classed as severe (patients with fluid deficit assessed to be greater than 10% of their bodyweight^5^), proportion of cases in children younger than 5 years, proportion of tested and positive rapid diagnostic tests, and proportion of tested and positive cultures. Exact binomial confidence intervals were calculated for all proportions.

**Figure 1:**
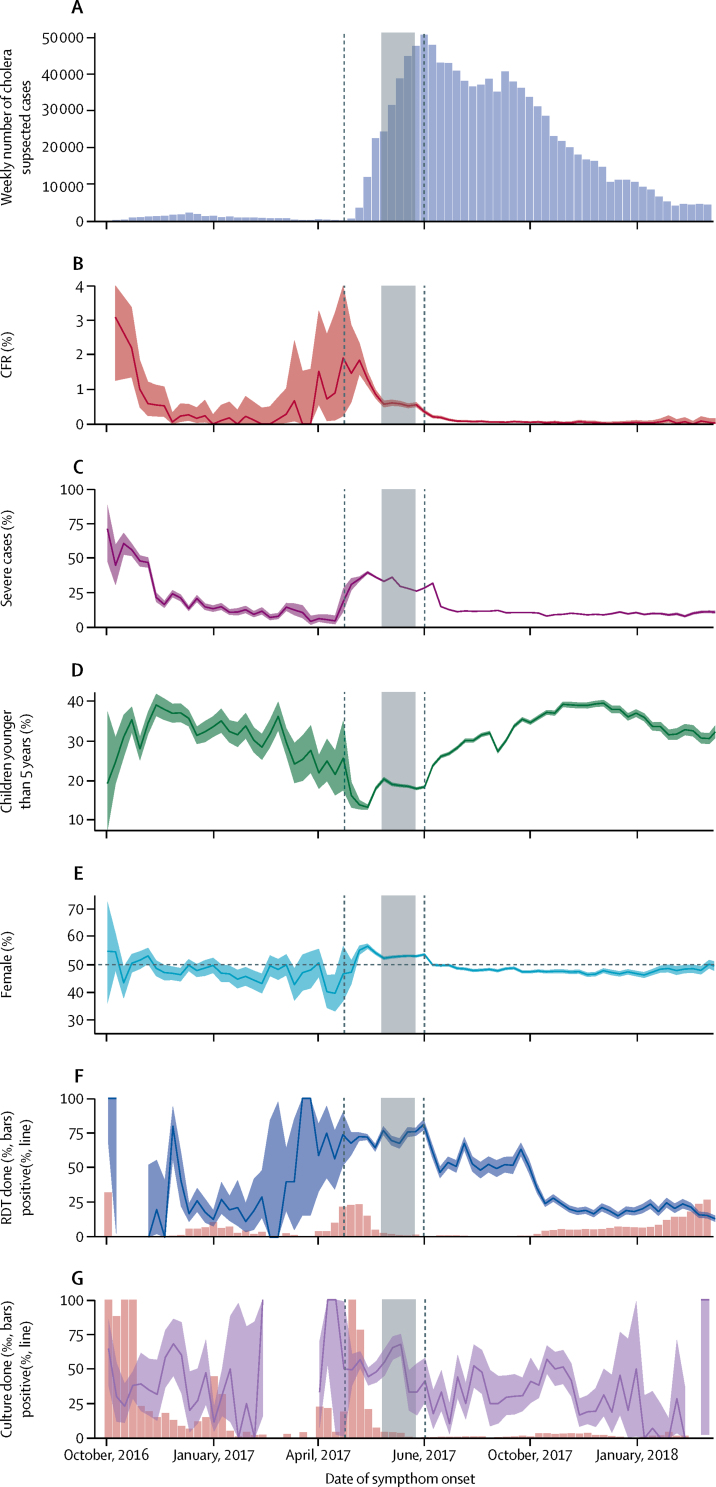
Weekly time series of key cholera indicators for Yemen between Sept 28, 2016, and March 12, 2018 (A) New suspected cholera cases. (B) Case fatality risk (CFR; number of deaths divided by number of suspected cases). (C) Proportion of severely dehydrated patients. (D) Proportion of cases in children younger than 5 years of age. (E) Proportion of female cases. (F) Percentage of suspected cases with a rapid diagnostic test (RDT; pink bars) and percentage positive (blue line). (G) Proportion of cases tested for culture confirmation (per 1000, pink bars) and percentage positive (purple line). Shaded areas correspond to exact binomial 95% CIs for proportions. The Ramadan period (May 26–June 24, 2017) is indicated by a grey rectangle. The first vertical dashed line defines the end of the first wave/start of the increasing phase of the second wave and the second vertical dashed line indicates the end of the increasing phase/start of the decreasing phase of the second wave of the epidemic.

To describe the spatiotemporal dynamics, we divided the epidemic into three time periods: the first wave (Sept 28, 2016, to April 23, 2017), the increasing phase of the second wave (April 24 to July 2, 2017), and the decreasing phase of the second wave (July 3, 2017, to March 12, 2018; figure 1A). For each period, we calculated the attack rate ratio of each district as the ratio of the district to country-level attack rates. An attack rate ratio above 1 indicates that a district had a higher attack rate compared with the national average during that epidemic phase.

We quantified the transmission rate of cholera through time by estimating the daily instantaneous reproduction number (*R*_t_),^16^ defined as the average number of secondary cases caused by an infected individual at time *t*. We used the daily suspected case counts and the serial interval distribution to estimate the posterior distribution of *R*_t_ using the R package EpiEstim^16^ (appendix pp 5–6). Consistent with previous estimates, we modelled the serial interval as a gamma distribution with a mean of 5 days and SD of 8 days.^17^

We estimated the association between rainfall and incidence of cholera during the increasing phase of the second wave (April 15–June 24, 2017). We fitted a quasi-Poisson generalised additive mixed model to the time series of suspected cases of the 285 districts that reported at least one cholera case during that period. For each day, we defined the accumulated rainfall over the previous 7 days as AR7D. We used a distributed-lag non-linear model with penalised splines^18^ to infer the cumulative effect of AR7D on cholera incidence over the following 10 days. We also tested whether the risk of becoming infected with cholera changed during Ramadan, when human movement and eating patterns tend to change, by including a fixed effect and a random effect for each district for the period May 26–June 24, 2017. The model also accounted for spatial clustering and changes in the immune population, as well as autocorrelation and overdispersion in the case counts (appendix pp 6–13).

We conducted two supplementary analyses in order to assess the risk of a third epidemic wave in 2018. First, we used results from rapid diagnostic tests and culture performed during the last 3 weeks of available data (from Feb 20, 2018 to March 12, 2018) in order to infer in which districts cholera transmission was likely to be still ongoing at that time (appendix pp 14–15). Second, we sought to make simple crude estimates of the proportion of the population that remained at risk in case of cholera re-introduction in 2018 (appendix pp 15–17). In brief, we tested six different scenarios by combining assumptions regarding the proportion of cholera infections reported up to March 12, 2018 (20% or 50%) and the expected basic reproduction number (R0) at the outset of a potential third wave (1·2, 1·6, or 2).

### Role of the funding sources

The Yemen Health Authorities, with support from WHO, set up the data collection system. The funders of the study had no role in study design, data analysis, data interpretation, or writing of the report. The corresponding author had full access to all the data in the study and had final responsibility for the decision to submit for publication.

## Results

Between Sept 28, 2016, and March 12, 2018, 1 103 683 suspected cholera cases and 2385 deaths were reported through the cholera surveillance system, representing an overall attack rate of 3·69% and a CFR of 0·22% (table 1). The epidemic had two waves, the first peaking in mid-December, 2016 (1935 weekly suspected cases), followed by a second larger wave from late April, 2017, which peaked at the end of June, 2017 (50 832 weekly suspected cases; figure 1A). During the 4 weeks preceding the start of the second wave, 998 suspected cholera cases were reported in 11 governorates. Five of these 11 governorates confirmed the cases by culture (figure 1).

**Table 1:** Cholera indicators by governorate and nationally for the first and second waves

	**Population**	**Cases**	**AR (%)**	**Deaths**	**CFR (%)**	**Rapid diagnostic test**		**Culture confirmation**	
						Tested, n (%*)	Positive, n (%†)	Tested, n (%*)	Positive, n (%†)
Abyan	611 303	29 057	4·75%	35	0·12%	312 (1·1%)	158 (50·6%)	115 (0·4%)	33 (28·7%)
Aden	956 667	22 635	2·37%	81	0·36%	688 (3·0%)	369 (53·6%)	230 (1·0%)	161 (70·0%)
Al Bayda	770 151	34 537	4·48%	47	0·14%	2182 (6·3%)	907 (41·6%)	200 (0·6%)	15 (7·5%)
Al Dhale'e	753 361	48 672	6·46%	85	0·17%	380 (0·8%)	130 (34·2%)	20 (0·0%)	7 (35·0%)
Al Hudaydah	3 345 560	163 533	4·89%	301	0·18%	4443 (2·7%)	1296 (29·2%)	411 (0·3%)	238 (57·9%)
Al Jawf	648 754	16 139	2·49%	22	0·14%	1515 (9·4%)	919 (60·7%)	36 (0·2%)	12 (33·3%)
Al Maharah	162 385	1168	0·72%	1	0·09%	263 (22·5%)	118 (44·9%)	21 (1·8%)	14 (66·7%)
Al Mahwit	760 725	64 247	8·45%	153	0·24%	2198 (3·4%)	1036 (47·1%)	71 (0·1%)	14 (19·7%)
Amanat Al Asimah	3 308 478	105 693	3·19%	73	0·07%	6251 (5·9%)	2377 (38·0%)	593 (0·6%)	204 (34·4%)
Amran	1 529 834	104 710	6·84%	178	0·17%	2499 (2·4%)	730 (29·2%)	116 (0·1%)	26 (22·4%)
Dhamar	2 121 016	104 888	4·95%	166	0·16%	3889 (3·7%)	491 (12·6%)	149 (0·1%)	34 (22·8%)
Hajjah	2 474 661	123 658	5·00%	443	0·36%	869 (0·7%)	230 (26·5%)	55 (0·0%)	23 (41·8%)
Ibb	3 065 230	71 281	2·33%	301	0·42%	905 (1·3%)	539 (59·6%)	107 (0·2%)	32 (29·9%)
Lahj	1 052 545	25 448	2·42%	23	0·09%	1228 (4·8%)	602 (49·0%)	82 (0·3%)	27 (32·9%)
Marib	359 586	7296	2·03%	7	0·10%	197 (2·7%)	128 (65·0%)	2 (0·0%)	0
Moklla	799 268	568	0·07%	2	0·35%	105 (18·5%)	80 (76·2%)	43 (7·6%)	32 (74·4%)
Raymah	633 758	19 188	3·03%	123	0·64%	639 (3·3%)	420 (65·7%)	41 (0·2%)	15 (36·6%)
Sa'ada	890 273	10 711	1·20%	5	0·05%	920 (8·6%)	752 (81·7%)	12 (0·1%)	0
Sana'a	1 250 811	79 132	6·33%	135	0·17%	3513 (4·4%)	873 (24·9%)	272 (0·3%)	66 (24·3%)
Say'on	668 880	23	0·00%	0	0·00%	22 (95·7%)	9 (40·9%)	8 (34·8%)	0
Shabwah	646 685	1484	0·23%	3	0·20%	128 (8·6%)	54 (42·2%)	20 (1·3%)	9 (45·0%)
Taizz	3 059 408	69 615	2·28%	201	0·29%	1648 (2·4%)	1006 (61·0%)	425 (0·6%)	325 (76·5%)
Yemen (first wave‡)	29 932 971	25 839	0·09%	120	0·46%	954 (3·7%)	251 (26·3%)	491 (1·9%)	181 (36·9%)
Yemen (second wave, increasing§)	29 932 971	277 167	0·93%	1663	0·60%	8154 (2·9%)	5873 (72·0%)	1286 (0·5%)	681 (53·0%)
Yemen (second wave, decreasing¶)	29 932 971	800 677	2·67%	602	0·08%	25 686 (3·2%)	7100 (27·6%)	1252 (0·2%)	425 (33·9%)
Yemen (total)	29 932 971	1 103 683	3·69%	2385	0·22%	34 794 (3·2%)	13 224 (38·0%)	3029 (0·3%)	1287 (42·5%)

AR=attack rate. CFR=case fatality risk. Socotra is the only Governorate that did not report any case, and was therefore not added to this table.

* Proportion of suspected cases that were tested (cholera surveillance system recommendation is 10% for rapid diagnostic test).

† Proportion of tested cases with positive result.

‡ From Sept 28, 2016, to April 23, 2017.

§ From April 24 to July 2, 2017.

¶ From July 3, 2017, to March 12, 2018.

The CFR only exceeded 1% in the early stages of the first and second epidemic waves (figure 1B), except in cases older than 65 years (overall CFR >1%; appendix p 2). The proportion of severe cases followed a similar trend (figure 1C). Children younger than 5 years accounted for 29% of the total suspected cases (table 2), but this proportion increased from 15% to 40% during the second epidemic wave (figure 1D). The overall sex ratio was 1·02, although there was a greater proportion of females among the suspected cases during the increasing phase of the second wave (figure 1E, table 2), in particular in those older than 15 years of age (58%, appendix p 3). 984 339 (96%) of 1 025 802 suspected cholera cases visited health facilities within 2 days of symptom onset (table 2).

**Table 2:** Patients' characteristics for the first and second waves

	First wave*	**Second wave**		**First and second waves**
		Increasing phase†	Decreasing phase‡	
**Suspected cholera cases, n (%)**				
<5 years	8509 (32·9%)	50 800 (18·3%)	256 423 (32·0%)	315 732 (28·6%)
≥5 years	17 075 (66·1%)	225 018 (81·2%)	534 361 (66·7%)	776 454 (70·4%)
Missing age	255 (1·0%)	1349 (0·5%)	9893 (1·2%)	11 497 (1·0%)
Total	25 839 (100%)	277 167 (100%)	800 677 (100%)	1 103 683 (100%)
**Deaths, n (%)**				
<5 years	42 (35·0%)	252 (15·2%)	159 (26·4%)	453 (19·0%)
≥5 years	74 (61·7%)	1395 (83·9%)	438 (72·8%)	1907 (80·0%)
Missing age	4 (3·3%)	16 (1·0%)	5 (0·8%)	25 (1·0%)
Total	120 (100%)	1663 (100%)	602 (100%)	2385 (100%)
**Case fatality risk (%)**				
<5 years	0·49%	0·50%	0·06%	0·14%
≥5 years	0·43%	0·62%	0·08%	0·25%
Missing age	1·57%	1·19%	0·05%	0·22%
Total	0·46%	0·60%	0·08%	0·22%
**Severe cases (%)**				
<5 years	1076/6129 (17·6%)	13 088/46 281 (28·3%)	22 806/250 418 (9·1%)	36 970/302 828 (12·2%)
≥5 years	2245/11 855 (18·9%)	64 131/206 400 (31·1%)	68 516/519 067 (13·2%)	134 892/737 322 (18·3%)
Missing age	42/145 (29%)	536/1018 (52·7%)	1049/9671 (10·8%)	1627/10 834 (15%)
Total	3 363/18 129 (18·6%)	77 755/253 699 (30·6%)	92 371/779 156 (11·9%)	173 489/1 050 984 (16·5%)
**Female (%)**				
<5 years	3 790/8 507 (44·6%)	23 190/50 800 (45·6%)	116 130/256 423 (45·3%)	143 110/315 730 (45·3%)
≥5 years	8451/17 075 (49·5%)	123 897/225 017 (55·1%)	264 780/534 361 (49·6%)	397 128/776 453 (51·1%)
Missing age	139/255 (54·5%)	748/1349 (55·4%)	4743/9893 (47·9%)	5630/11 497 (49%)
Total	12 380/25 837 (47·9%)	147 835/277 166 (53·3%)	385 653/800 677 (48·2%)	545 868/1 103 680 (49·5%)
**Positive rapid diagnostic test, n/N (%)**				
<5 years	44/208 (21·2%)	629/966 (65·1%)	1245/6021 (20·7%)	1918/7195 (26·7%)
≥5 years	206/743 (27·7%)	5222/7153 (73·0%)	5790/19 428 (29·8%)	11 218/27 324 (41·1%)
Missing age	1/3 (33·3%)	22/35 (62·9%)	65/237 (27·4%)	88/275 (32·0%)
Total	251/954 (26·3%)	5873/8154 (72·0%)	7100/25 686 (27·6%)	13 224/34 794 (38·0%)
**Positive culture, n/N (%)**				
<5 years	31/143 (21·7%)	21/52 (40·4%)	47/147 (32·0%)	99/342 (28·9%)
≥5 years	146/336 (43·5%)	651/1213 (53·7%)	370/1043 (35·5%)	1167/2592 (45·0%)
Missing age	4/12 (33·3%)	9/21 (42·9%)	8/62 (12·9%)	21/95 (22·1%)
Total	181/491 (36·9%)	681/1286 (53·0%)	425/1252 (33·9%)	1287/3029 (42·5%)
**Time to admission, n/N (%)**				
Same day	4352/17 998 (24·2%)	78 189/219 926 (35·6%)	249 499/787 878 (31·7%)	332 040/1 025 802 (32·4%)
1 day	8242/17 998 (45·8%)	124 949/219 926 (56·8%)	455 314/787 878 (57·8%)	588 505/1 025 802 (57·4%)
2 days	1491/17 998 (8·3%)	8176/219 926 (3·7%)	54 127/787 878 (6·9%)	63 794/1 025 802 (6·2%)
>2 days	3913/17 998 (21·7%)	8612/219 926 (3·9%)	28 938/787 878 (3·7%)	41 463/1 025 802 (4%)

* From Sept 28, 2016, to April 23, 2017.

† From April 24 to July 2, 2017.

‡ From July 3, 2017, to March 12, 2018.

**Figure 2:**
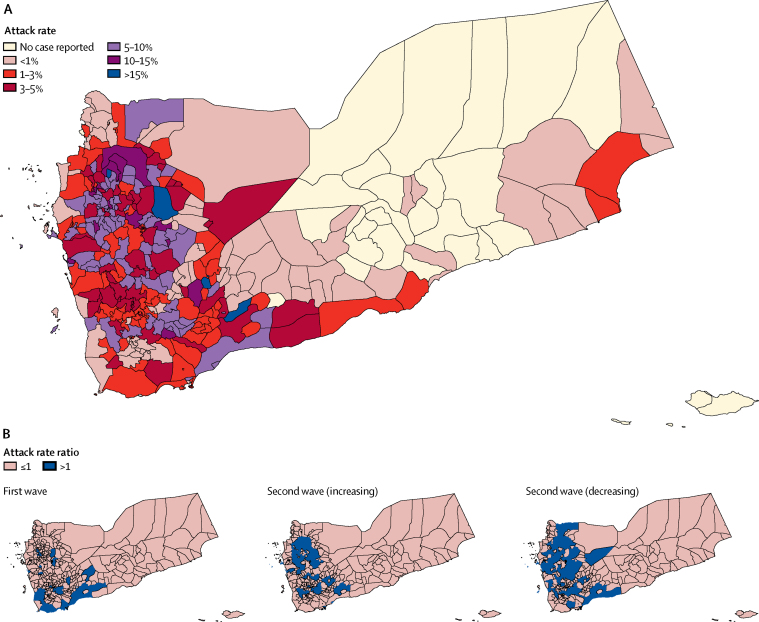
Spatial distribution of suspected cholera cases (A) Attack rate by district between Sept 28, 2016, and March 12, 2018. (B) Attack rate ratio by district for the first wave (Sept 28, 2016, to April 23, 2017) and increasing phase (April 24 to July 2, 2017) and decreasing phase (July 3, 2017, to March 12, 2018) of the second wave of the epidemic.

305 (92%) of 333 districts, mainly in the western part of Yemen, reported cases with highly heterogeneous attack rates (figure 2A, table 1). The spatial distribution of cases was distinct during each of the three time periods considered (figure 2B). Among the 180 districts affected during the first epidemic wave, southern districts were most at risk, although some northern districts also had an attack rate ratio above 1. The increasing phase of the second wave predominantly affected northern and central districts, located in the mountainous part of Yemen (appendix p 4), before expanding to coastal districts during the decreasing phase. The second wave showed more rapid geographical expansion: the number of districts reporting cases increased from six to 57 within 4 weeks during the first wave; and from 19 to 213 during the second wave, 163 (77%) of which were also affected during the first epidemic wave (appendix p 5).

Between Sept 28, 2016, and March 12, 2018, 1287 (43%) of the 3029 samples were confirmed as *V cholerae* O1 serotype Ogawa by culture (figure 1G, table 1). Additionally, 13 224 (38%) of 34 794 suspected cases tested positive by rapid diagnostic test. However, this percentage varied over time, rising to 81% at the peak of the second wave (figure 1F).

Molecular analyses showed that all 41 strains (ten isolated in 2016, 31 in 2017) were El Tor biotype (*rstR*^ElTor^), shared the same *V cholerae* O1 variant strains *ctxB* genotype and *tcpA* sequence and the same antibiotic resistance phenotype, in particular resistance to nitrofurantoin, trimethoprim, and nalidixic acid, as well as a decreased sensitivity to fluoroquinolones. These common phenotypic and genotypic characteristics suggest that a single strain circulated during both waves in Yemen. Additional whole genome sequence analyses are being done at the Pasteur Institute (Paris, France) to conclusively confirm these results.

**Figure 3:**
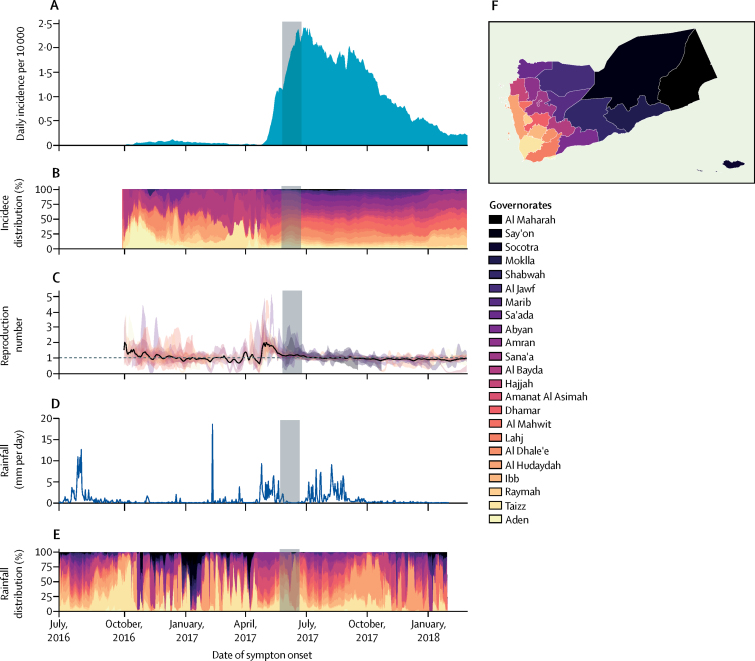
Daily time series of incidence, reproduction number, and rainfall by governorate between July 1, 2016, and March 12, 2018 (A) National incidence. (B) Contribution of each governorate to the national incidence. (C) Time-varying instantaneous reproduction number *R*_t_ represented by the mean estimate for the country (black line) and 95% credible interval for each governorate (shaded areas). (D) Country-level rainfall (mm per day). (E) Contribution of each governorate to the country rainfall. To obtain meaningful rainfall time series for comparison with cholera incidence time series at the national and governorate levels, we used a weighted mean of the district level rainfall time series, with daily weights proportional to the number of cases reported in each district over the following 2 weeks. To smooth the high level of noise in the daily reporting of suspected cases, we performed a rolling average with a 5-day time window on both the incidence (A) and reproduction number (C) time series. The Ramadan period (May 26–June 24, 2017) is indicated by a grey rectangle.

The first suspected cases were reported from four governorates in the final week of September, 2016, at the end of the rainy season (appendix p 5). Cholera then spread to 17 governorates with an overall *R*_t_ above the epidemic threshold of 1 (figure 3). The first wave decreased at the end of 2016, and despite some localised resurgence, *R*_t_ remained below 1 for the dry season, which ended in April, 2017.

As the rains returned, *R*_t_ rapidly increased nationally to 2 with 13 of 23 governorates having a median *R*_t_ of more than 2. These reproduction numbers translated into an increase in national daily incidence from 0·01 to 1 per 10 000 between April 15 and May 15, 2017 (figure 3A). At the national level, *R*_t_ dropped during the second half of May, which coincided with the end of the spring rains, stabilising around 1–2. This period coincided with Ramadan (May 26–June 24, 2017), during which the daily incidence further increased to 2·4 cases per 10 000. The epidemic peaked 1 week after the end of Ramadan.

As the summer rainy season started, *R*_t_ again stabilised around 1 at the national level although in some governorates, such as Al Hudaydah, incidence increased again following the second peak of rainfall in August. Coinciding with the end of the summer rains, in October, 2017, *R*_t_ dropped below 1 and the daily incidence returned to less than 1 per 10 000.

**Figure 4:**
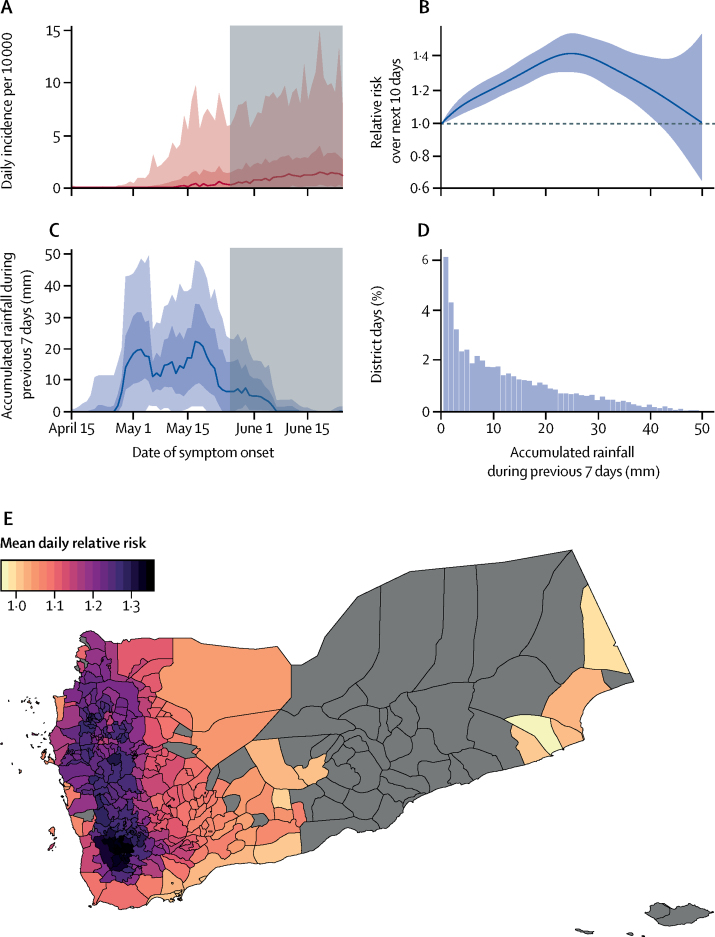
Detailed analysis of the effect of rainfall on cholera incidence during the increasing phase of the second epidemic wave (April 15–June 24, 2017) (A) Daily incidence and (C) accumulated rainfall during the 7 previous days (AR7D) in mm. Solid lines represent the day-wise median over all districts. Dark and light shaded areas represent the IQR and 95% quantile intervals (centred on the median), respectively. The Ramadan period (May 26–June 24, 2017) is indicated by a grey rectangle. (B) Relative risk (the ratio of cholera risk for individuals, cumulated over 10 days after a given AR7D exposure, to the risk when unexposed). Shaded area represents 95% CI. (D) AR7D distribution for all districts and days. The proportion for 0 mm is equal to 46% (omitted for the sake of visibility). (E) Mean daily relative risk attributable to rainfall during the rainy season (districts with no cases reported are in grey). For each district, the baseline risk corresponds to a typical day following a week with no rain. See appendix p 10 for a map of the highest daily relative risk.

The median cumulative rainfall over the previous 7 days rapidly increased to 20 mm at the end of April and remained between 10 mm and 30 mm, before dropping at the end of the third week of May, 2017 (figure 4C and 4D). We assessed rainfall as a driver of the expansion phase of the second wave (figure 4A).

The relative risk of being reported as a suspected cholera case in the 10 days following a week with 10 mm of cumulative rainfall was 1·21 (95% CI 1·15–1·28) compared with a week with no rain (figure 4B). Precisely, we estimated a non-linear rainfall-incidence risk response curve, with a peak at AR7D 25 mm, corresponding to a relative risk of 1·42 (95% CI 1·31–1·55) compared with no rain. The model showed a good fit to the data (*R*^2^=0·72, appendix p 11) and explained 80% of the deviance. In sensitivity analyses, models with different assumptions about the accumulated rainfall and lag periods showed qualitatively similar results (appendix pp 11–12).

We found substantial district-level heterogeneity in the effect of rainfall (figure 4E). The districts most at risk are on the mountainous north-south axis from Sa'ada to Taizz with coastal districts being at lower risk. Districts in the central governorates of Ibb and Taizz and in the northern governorate of Hajjah had the highest mean daily relative risk (>1·3) during the rainy period, from April 24 to June 1, 2017.

Finally, we found that the risk of being reported as a suspected cholera case during Ramadan was 1·19 (95% CI 1·14–1·25) times higher than the risk during the preceding month and that this risk varied significantly across districts (appendix pp 12–13).

Results from 472 of 3119 positive rapid diagnostic tests and two positive cultures performed over the last 3 weeks of available data revealed that, as of March 12, 2018, cholera transmission was still active in 11 districts located in the Governorates of Ibb, Amanat Al Asimah, Al Hudaydah, Al Jawf, Al Bayda, Sana'a, and Al Mahwit. However, there were not enough rapid diagnostic tests or cultures performed in 131 districts in order to conclude whether transmission was still ongoing or had already stopped (appendix pp 14–15).

Under the most conservative scenario (i.e. the one that would lead towards lower estimates of the epidemic risk) assuming a reporting rate of 20% in 2016 and 2017 and a R0 of 1·2 in 2018, we found that 54% of the districts were at risk of an epidemic in case of cholera re-introduction in 2018, totalling a population at risk of more than 13·8 million, which represents almost 50% of the population in the 305 districts previously affected by the first and second waves (appendix pp 15–17).

## Discussion

Our results suggest that the spring rainy season triggered the large second wave of cholera in Yemen in late April, 2017. The second wave occurred synchronously across districts in Yemen, most of which had been affected during the first wave. The period of highest cholera transmission was limited to the 4 weeks of the spring rainy season, during which the daily national cholera incidence increased by 100 times and cholera spread across the entire country. By comparison, the summer rainy season in July–August had a smaller impact on transmission, mainly prolonging the second wave. This reduced effect possibly resulted from the scale-up of water, sanitation, and hygiene (WASH) interventions, which tripled between the spring and summer rainy seasons.^19^ These observations suggest that the transition between the dry and rainy seasons in April is key for WASH interventions in Yemen.

Previous work has mechanistically connected variation in rainfall to cholera transmission through decreased water levels during drought leading to increased use of unsafe water sources;^20^ contamination of water sources by flooding;^20^, ^21^ and synergistic effects with zooplankton in waterways, increased iron in water leading to higher *V cholerae* survival, or variations in the bacteriophage population.^22^ Some of these mechanisms are plausible in the context of this epidemic. Yemen is affected by water scarcity, with most water drawn from deep groundwater.^4^ The falling water table and fuel crisis resulting from conflict have made groundwater extraction more expensive,^3^ possibly resulting in increased use of surface water during the rainy season, especially at its start in April, when water resources are at their lowest after 6 months of droughts. When wastewater and solid waste management systems are damaged, as they have been during the Yemen conflict, they can easily contaminate surface water.^23^, ^24^ Further investigation of the mechanistic link between rainfall and cholera transmission in Yemen is needed, but this should not prevent the enhancement of current control efforts to reduce risk during the upcoming rainy season, for instance through point-of-use or household water filtration and disinfection.^25^

We found that the period of Ramadan that followed the spring rains was also associated with an increased risk of cholera transmission, suggesting that social behaviour could have influenced the epidemic spread in Yemen. During Ramadan, there is an increase in large gatherings for meals in which people share food,^26^ especially for Iftar (the daily breaking of fast at sunset), and people more frequently eat food from street vendors, which has been associated with an increased risk of cholera transmission in other settings.^27^ These findings suggest that a large-scale campaign for hygiene education and public health information should be implemented during the 2018 Ramadan (May 15 to June 14), possibly involving Imams to deliver these messages.

Our study is based on a well maintained line-list database of more than 1 million records that allowed us to estimate the time-varying reproduction number for each governorate, and to quantify the increased cholera risk following the rains in April, 2017. However, our analysis of the drivers of transmission in the second wave are based on a single cholera season and our estimates should be interpreted with caution. If cholera transmission in Yemen continues, this association should be reassessed, ideally using rainfall data measured by ground weather stations (currently not available in Yemen) rather than estimated from satellite imagery, which often underestimates precipitation events (appendix p 13).^15^ Furthermore, our model did not account for long range population movements, which certainly had an important role in the spread of cholera, nor for changes in cholera surveillance over time, which could have affected the shape of the epidemic curve.

Although the case definition for suspected cholera is highly sensitive, it is not specific, and can lead to misclassification of diarrhoea due to other causes. Additionally, poor adherence to the case definition might have happened in inexperienced health facilities. More specific indicators such as the percentage of positive rapid diagnostic tests, the percentage of severe cases, and the proportion of patients younger than 5 years of age (who are more frequently affected by other enteric pathogens^28^) suggest that an increasing proportion of non-cholera diarrhoea cases could have been recorded in the cholera surveillance system during the decreasing phase of the second wave.

Over-reporting of cases has likely contributed to underestimates of the CFR in this epidemic. Additionally, poor access to health facilities, especially in areas near the frontline and directly affected by the war, could have led to underestimation of cholera burden, notably of mortality, as previously demonstrated in Haiti.^29^ However, Médecins Sans Frontières' experience in Yemen suggests that the low estimates of the CFR could also reflect high rates of health-care seeking among the Yemeni population. Nearly all patients (96%) visiting health facilities with suspected cholera arrived within 2 days of symptom onset. Unlike in other countries where cholera occurs, the first places people in Yemen visited for care tended to be health facilities taking part in the cholera surveillance system, as opposed to private clinics or non-allopathic healers. Until reliable cholera community death estimates become available for Yemen, CFR estimates and the accompanying confidence intervals should be interpreted with caution.

If the relation between rainfall and cholera incidence is causal, the probability of observing another epidemic wave increases with both the magnitude of the 2018 rainy season and the fraction of individuals remaining susceptible in each district. Although naturally acquired immunity following exposure to cholera is well established,^30^ the proportion of susceptible individuals depends on the duration and level of protection in symptomatic and asymptomatic infections, which is still unclear.^31^ However, on the basis of conservative assumptions, it is unlikely that the proportion of susceptible individuals is sufficiently low to reduce the reproduction number below the epidemic threshold and prevent future transmission in Yemen (appendix pp 15–17). Therefore, reducing the susceptible population might be an effective way to interrupt transmission or at least reduce the likelihood of widespread transmission when the rains return. Vaccination could achieve this reduction in the risk of cholera resurgence.^32^

To improve the chances of detection and containment in areas likely to be affected by ongoing transmission, the coverage of microbiological testing of suspected cases should be increased. As of March 2018, microbiological evidence suggests that there is both insufficient testing in 131 districts but, importantly, continuing transmission in 11 highly populated, mostly urban, districts (appendix pp 14–15). These urban districts would therefore benefit from immediate control efforts in order to mitigate the risk of cholera during the 2018 rainy season.

In conclusion, we found that the cholera epidemic in Yemen was shaped by the timing between cholera introduction, in September, 2016, and emergence of favourable environmental conditions for cholera transmission in April, 2017. The small first wave seeded cholera across the country during the dry season, leading to amplification in a major second wave during the rainy season. These findings have operational implications, including the need for improvements in epidemiological and laboratory surveillance, vaccination, and water and sanitation interventions. If localised resurgence of cholera cases can rapidly be detected and contained during the 2018 rainy season, a potential third wave could be avoided. We thus make an urgent call for action on the part of local officials, donors, and international partners, to mitigate the risk of a new cholera epidemic wave in Yemen, which would certainly further weaken a highly vulnerable population.
