# Supplementary material for: Cholera epidemic in Yemen, 2016–18: an analysis of surveillance data
Source: Lancet Glob Health. 2018 May 3;6(6):e680–90. doi: 10.1016/S2214-109X(18)30230-4 (PMC5952990; doi:10.1016/S2214-109X(18)30230-4)
Supplement: Supplementary appendix [file mmc1.pdf]

THE LANCET
Global Health

Supplementary appendix

This appendix formed part of the original submission and has been peer reviewed. We post it as supplied by the authors.

Supplement to: Camacho A, Bouhenia M, Alyusfi R, et al. Cholera epidemic in Yemen, 2016–18: an analysis of surveillance data. *Lancet Glob Health* 2018; published online May 3. http://dx.doi.org/10.1016/S2214-109X(18)30230-4.

Cholera epidemic in Yemen, 2016–18: an analysis of surveillance data

Appendix

Anton Camacho[1,2], Malika Bouhenia[3], Reema Alyusfi[4], Abdulhakeem Alkohlani[4], Munna Abdulla Mohammed Naji[5], Xavier de Radiguès[3], Abdinasir M. Abubakar[6], Abdulkareem Almoalmi[3], Caroline Seguin[7], Maria Jose Sagrado[8], Marc Poncin[9], Melissa McRae[10], Mohammed Musoke[11], Ankur Rakesh[1], Klaudia Porten[1], Christopher Haskew[12], Katherine E. Atkins[2], Rosalind M. Eggo[2], Andrew S. Azman[13], Marije Broekhuijsen[14], Mehmet Akif Saatcioglu[3], Lorenzo Pezzoli[12], Marie-Laure Quilici[15], Abdul Rahman Al-Mesbahy[5], Nevio Zagaria[3], Francisco J. Luquero[1]

1. Epicentre, Paris, France
2. London School of Hygiene & Tropical Medicine, London, UK
3. World Health Organization, Sana'a, Yemen
4. Health Authorities, Yemen
5. Central Public Health Laboratory, Sana'a, Yemen
6. World Health Organization, Cairo, Egypt
7. Médecins sans Frontières, Dubai, United Arab Emirates
8. Médecins sans Frontières, Barcelona, Spain
9. Médecins sans Frontières, Geneva, Switzerland
10. Médecins sans Frontières, Amsterdam, Netherlands
11. Médecins sans Frontières, Sana'a, Yemen
12. World Health Organization, Geneva, Switzerland
13. Johns Hopkins School of Public Health, Baltimore, USA
14. UNICEF, Sana'a, Yemen
15. National Reference Center for Vibrios and Cholera, Institut Pasteur, Paris, France

Corresponding author:

Anton Camacho
Address: Epicentre, 55 rue Crozatier, 75012 Paris, France
E-mail: anton.camacho@epicentre.msf.org
Tel: +33.1.40.21.57.62

Age-sex characteristics

Figure S1 shows the percentage of cholera cases and deaths per age-group compared with the distribution of the general population in Yemen.[1] Children cases under five years of age were over-represented during the epidemic and accounted for 28.6% of the total number of cases. Adults aged above 45 years of age were at increased risk of deaths and were over-represented in the total number of deaths.

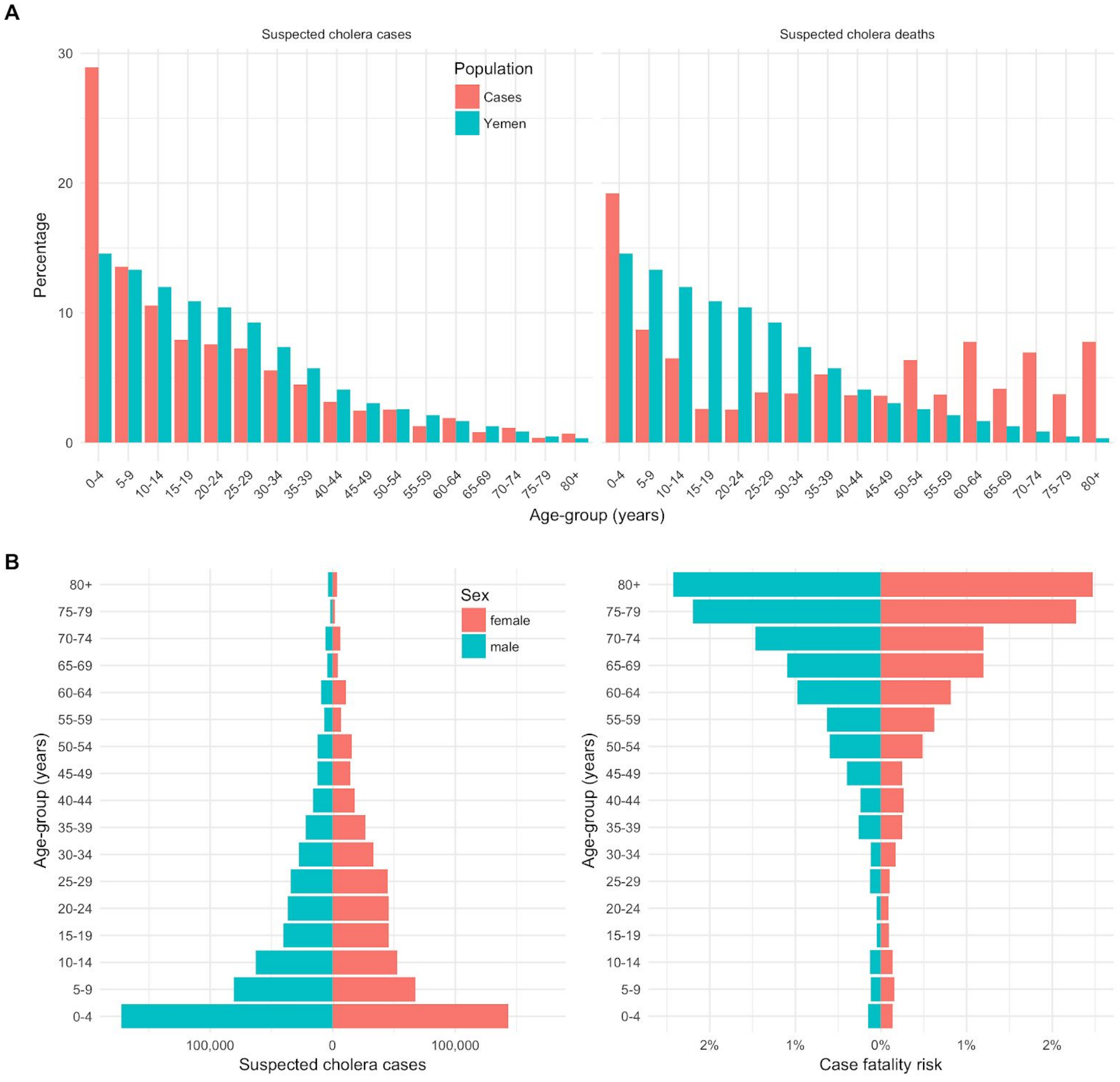

Figure S1: Age-sex distribution for number of cholera suspected cases, deaths and case fatality risk (September 28, 2016 to March 12, 2018).

Although the overall sex-ratio of cholera cases was equal to the sex-ratio in the population (1.02), we found some sex bias during the increasing phase of the second wave. We observed a significant over-representation of adult women, especially between 15 and 50 years of age, during the increasing phase of the second wave (Figure S2). This effect could be associated with a higher risk for women in age of childbearing, who could be potentially more involved in caregiving for cholera infected relatives at home. This sex bias could also result from differences in health seeking behaviours if women were more prone than men to seeking care.

All ages confounded, the peak of the national epidemic was observed early July 2017. However, when we restricted the analysis to children under 9 years of age, the epidemic peaked later, at the end of the summer rainy season, which could be related to diarrhea caused by other pathogens than *V. cholerae*.

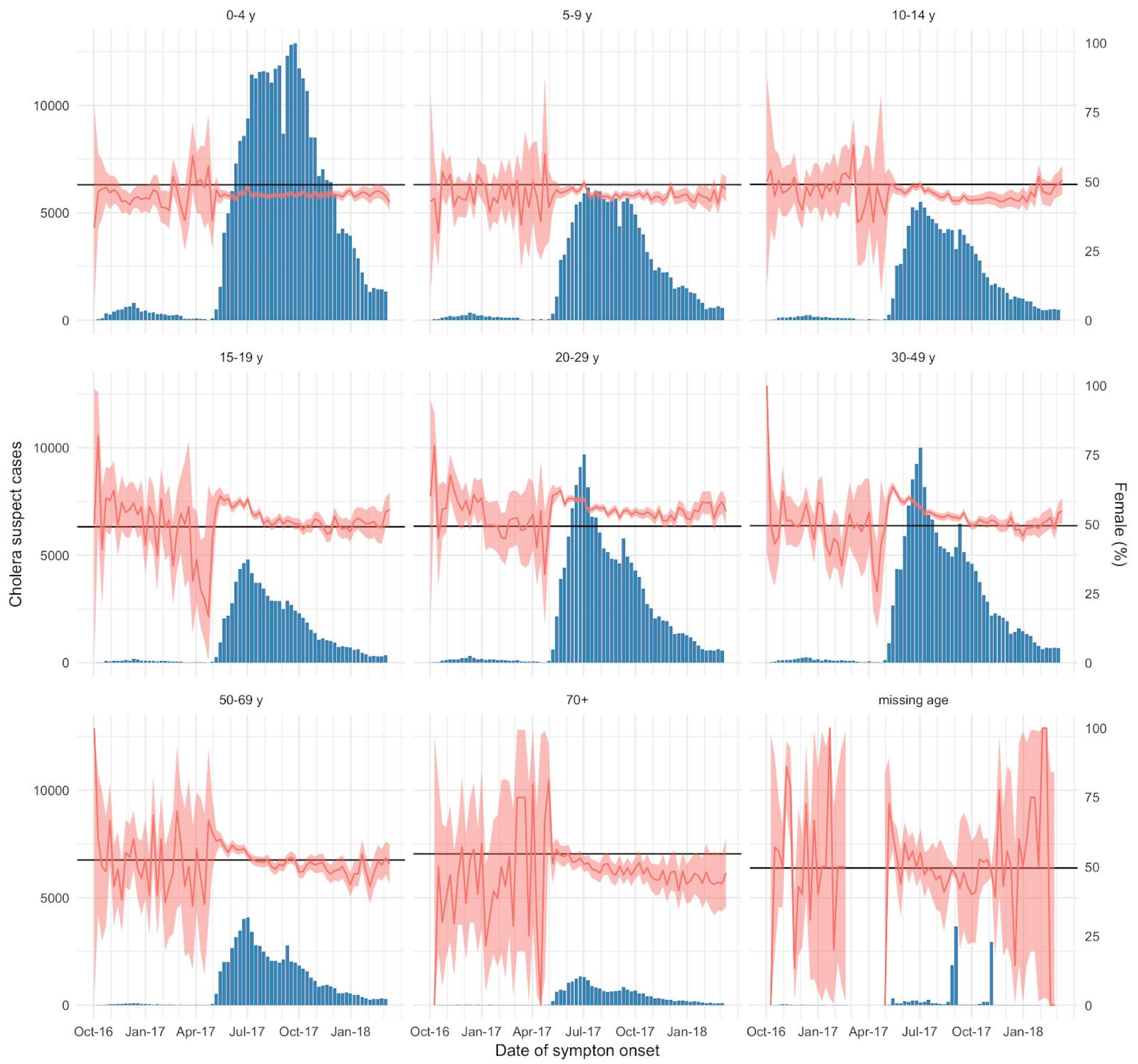

Figure S2: Time-series of the weekly number of cholera cases by age-group (blue bars) and the corresponding proportion of females (red line) with 95% exact binomial confidence intervals (red shaded envelope). The expected proportion of females in each age-group is indicated as an horizontal black line (source: UN World Population Prospects 2017 https://esa.un.org/unpd/wpp/)

Map of Yemen

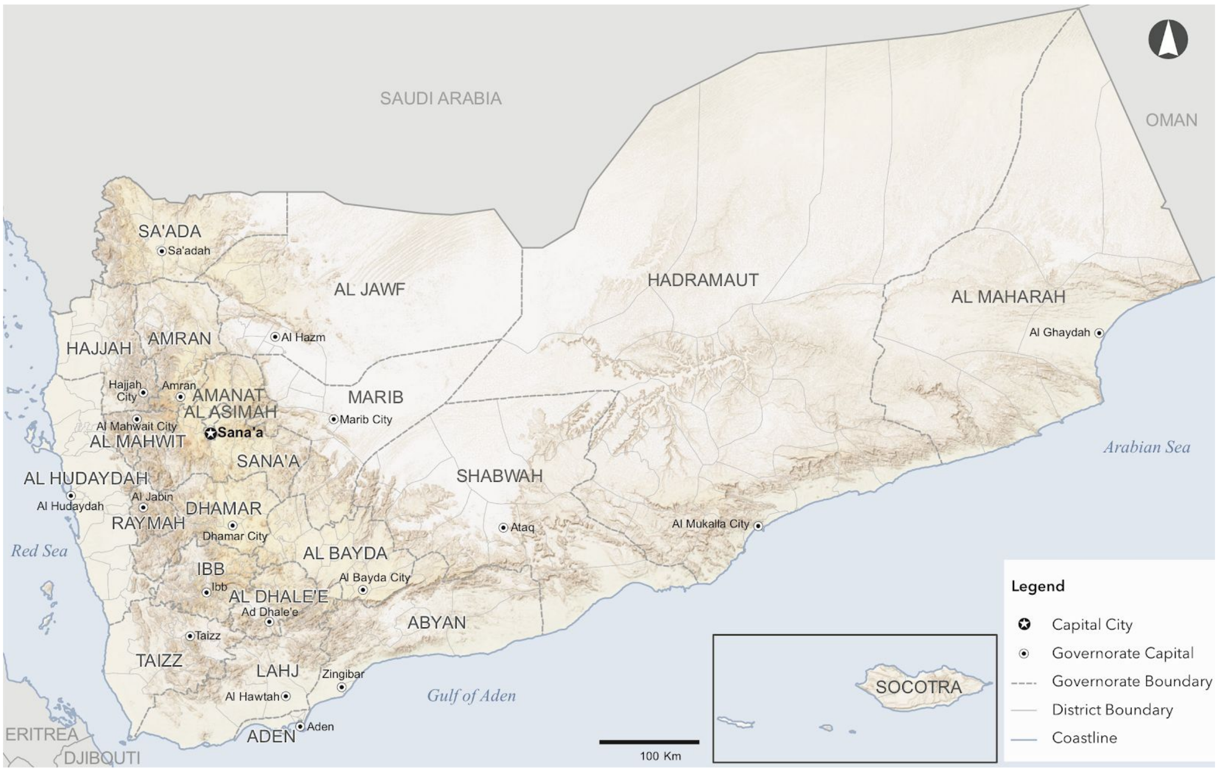

Figure S3: Yemen reference map extracted from UNOCHA' Humanitarian Needs Overview report.[2] The boundaries and names shown and the designations used on this map do not imply official endorsement or acceptance by the United Nations. Creation date: 20 October 2017. Sources: GoY/MoLA/CSO.

Location of cases reported in the first four weeks of both epidemic waves

Figure S3 shows the location of cases (centroid of district) reported in the first four weeks of both epidemic waves. During the first wave, which started at the beginning of the dry season, four Governorates reported cases during the first week, while during the second wave, which started at the beginning of the rainy season, seven Governorates reported cases. The graphs suggests that both the initial number of locations reporting cholera and the environmental triggering factors might explain the very distinct dynamic of both waves.

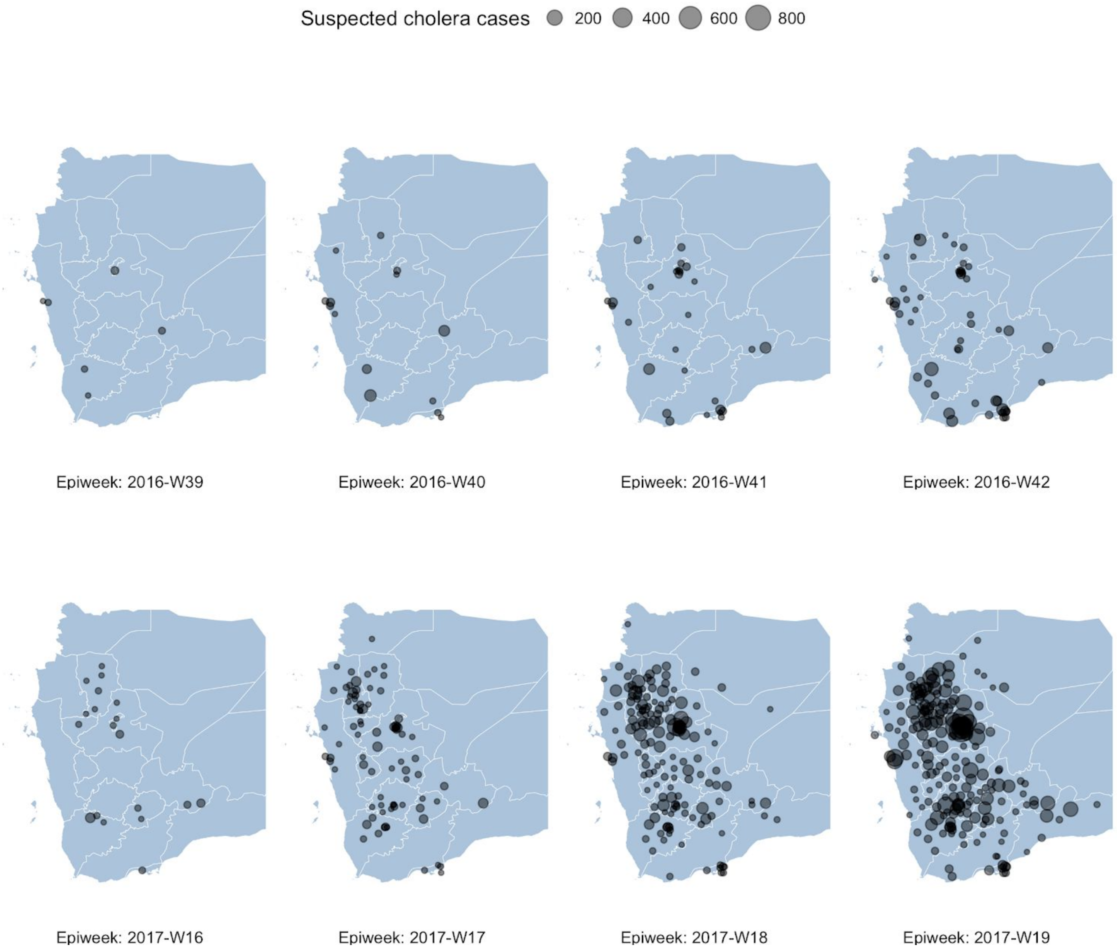

Figure S4: Geographical distribution of the number of suspected cholera cases during the first four weeks of the first (top panels) and second (lower panel) epidemic waves. Note: no cases were reported in in Eastern parts of the country excluded from the maps during these time periods.

National instantaneous reproduction number

In the main text, we estimated the instantaneous reproduction number, R_t, in each Governorate using a Bayesian framework (R package *EpiEstim*[3]). This method used the daily suspected case counts, the serial interval distribution and made the assumption that transmissibility was constant during a chosen time-window of 5 days

(equal to the mean serial interval). We then calculated the mean national estimate of R_t by averaging the mean estimates of each Governorate. By contrast, here we inferred R_t directly from the daily national case counts. Figure S5 shows that the direct estimate of R_t is similar to the indirect estimate of Figure 3. However, the 95% credible intervals are tight and do not reflect the variability observed across Governorates (Figure 3).

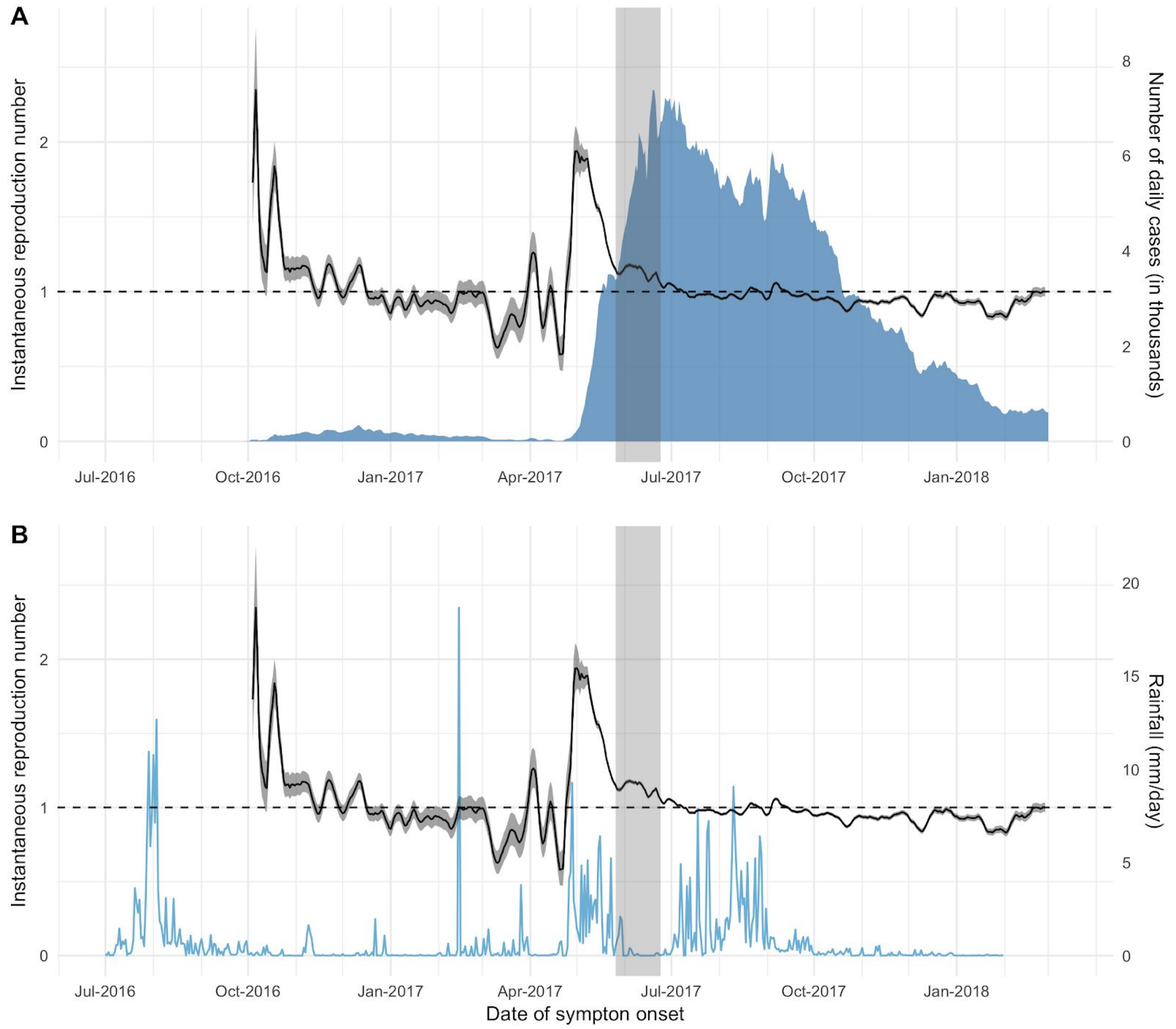

Figure S5: Time-series of daily incidence, reproduction number and rainfall. **A** Daily incidence per 10,000 and **B** daily rainfall at the national level. The time-varying instantaneous reproduction number R_t is super-imposed on both panels and represented by the mean estimate for the country (black line) and 95% credible intervals (shaded envelope).

Description, fit and sensitivity analysis for the regression model

We conducted a spatiotemporal analysis to quantify the association between different factors (e.g. rainfall, ramadan period) and cholera incidence during the increasing phase of the second epidemic wave: from April 15 to June 24, 2017. We restricted our analysis to the 285 districts that reported at least one suspected cholera case during that period (Figure S6).

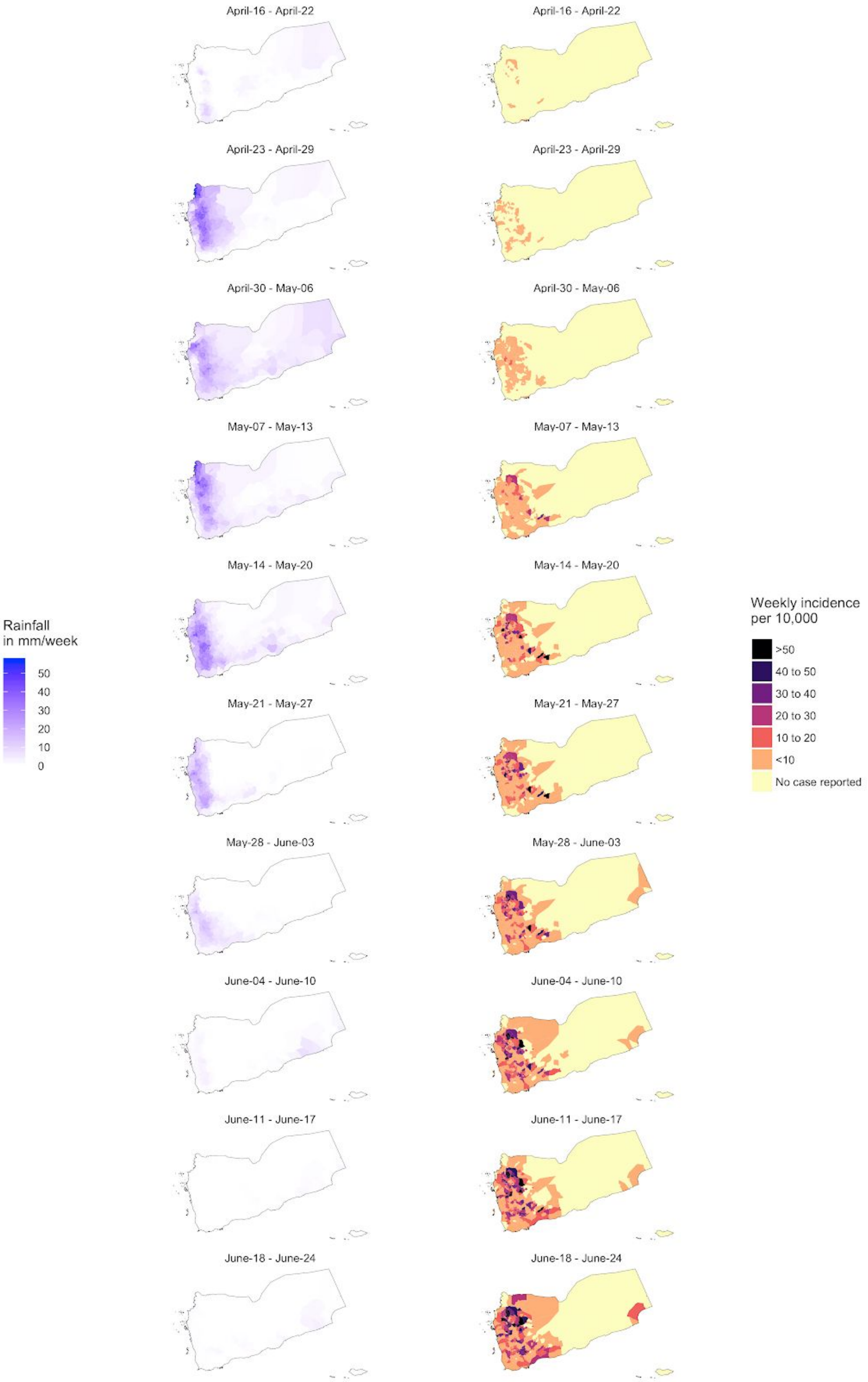

Figure S6: Spatiotemporal description of the rainfall and incidence by district and by week during the increasing phase of the second epidemic wave (April 15 2017 - June 26 2017).

Our approach builds-upon the methods used to estimate the posterior distribution of the instantaneous reproduction number in the main text (e.g. Figure 3 and Figure S5). This method assumes the daily number of cholera cases in district i, noted $I_{i,t}$, can be approximated with a Poisson process following the renewal equation:[4]

$$I_{i,t} \sim \text{Pois}\Big(R_{i,t} \sum_{s=0}^{t} I_{i,t-s}\omega_s\Big),$$

where $R_{i,t}$ is the instantaneous reproduction number in district i and ω is the discrete probability distribution of the serial interval (shifted Gamma distribution, with shift = 1 day, mean = 5 days and standard deviation = 8 days, consistent with previous works[5,6]).

First, one can make explicit the dependency of $R_{i,t}$ on the reduction of the number of susceptible caused by the epidemic:

$$R_{i,t} = \tilde{R}_{i,t}\frac{S_{i,t}}{N_i},$$

where $\tilde{R}_{i,t}$ can be seen as a basic instantaneous reproduction number (i.e. average number of secondary cases in a fully susceptible population), $S_{i,t}$ is the number of susceptible individuals and N_i is the population size (assumed to remain constant over time). $S_{i,t}$ is not observed but estimated from subtracting to N_i the cumulative sum of past incident cases:

$$S_{i,t} = N_i - \sum_{s=t_0}^{t-1} I_{i,s},$$

where t_0 is September 28, 2016, day of first suspected cholera case reported in Yemen. We therefore made the implicit assumption that all recovered cholera cases remained protected against reinfection for the entire epidemic. Since cholera immunity is believed to last for 5-10 years, this is a reasonable assumption for the time-frame of the Yemen epidemic (less than two years).

Replacing the new expression of $R_{i,t}$ in the renewal equation and taking the logarithm of the expectation of $I_{i,t}$, one obtains:

$$\log(\text{E}[I_{i,t}]) = \log(\tilde{R}_{i,t}) + \log\Big(\sum_{s=0}^{t} I_{i,t-s}\omega_s\Big) + \log\Big(1 - \frac{\sum_{s=t_0}^{t-1} I_{i,s}}{N_i}\Big)$$

This expression can be interpreted as a standard time-series Poisson regression model[7], with the right-hand side of the equality being composed of three terms: the first term for the transmissibility of cholera, the second for the force of infection and the final term for the depletion of the susceptible population (i.e. build-up of herd immunity). The parameters $\tilde{R}_{i,t}$ can be estimated using standard Poisson regression techniques.

Previous works[8,9] have used a similar model to link the transmissibility of cholera to rainfall in Dhaka (Bangladesh) by first estimating the transmissibility parameters for each time point using smoothing splines and then considering associations with rainfall (and other explanatory factors) in a second stage. By contrast, in our

analysis we used a direct incorporation of explanatory variables in a single stage by modelling $\log(\tilde{R}_{i,t})$ as follows:

$$\log(\tilde{R}_{i,t}) = \beta_0 + \sum_{d=1}^{6} \beta_{\text{day},d}\delta_{d,t} + \sum_{g=1}^{23} \beta_{\text{gov},g}\delta_{g,i} + (\beta_{\text{ram}} + \beta_{\text{ram},i})\delta_{\text{ram},t} + f_{\text{mrf}}(i) +$$
$$s(x_{i,t-l_0}, \ldots, x_{i,t-L}),$$

where:

- β_0 is the intercept

- $\beta_{\text{day},d}$ is a fixed effect for each day of the week to account for potential bias in reporting. $\delta_{d,t}$ is an indicator of day of the week (equal to 1 if time t is day of the week d and 0 otherwise). We use Monday as the reference day.

- $\beta_{\text{gov},g} \sim N(0, \sigma_{\text{gov}})$ is a random effect to account for unmeasured confounders at Governorate-level, such as heterogeneities in the reporting of cases to the national level. $\delta_{g,i}$ is an indicator of Governorate (equal to 1 if district i is in Governorate g and 0 otherwise).

- β_{ram} is the fixed effect for the period of Ramadan and $\beta_{\text{ram},i} \sim N(0, \sigma_{\text{ram}})$ is a random effect accounting for potential district heterogeneities. $\delta_{\text{ram},t}$ is an indicator of Ramadan (equal to 1 if time t is between May 26, 2017 and June 24, 2017, 0 otherwise).

- f_{mrf} models the district-level spatial heterogeneity using a Markov random field smoother that accounts for the neighbouring structure of the districts.

- $s(x_{i,t-l_0}, \ldots, x_{i,t-L})$ models the association between rainfall and cholera incidence.

More precisely, we modelled the association between weekly rainfall and cholera incidence during the following 10 days using a penalized distributed lag non-linear model (DLNM), which has been described in details elsewhere.[10,11]

In brief, the DLNM can describe complex non-linear and lagged dependencies through the function s - also called cross-basis - and defined as:

$$s(x_{i,t-l_0}, \ldots, x_{i,t-L}) = \sum_{l=l_0}^{L} f.w(x_{i,t-l}, l),$$

where $x_{i,t-l}$ is the accumulated rainfall over the 7 previous days (AR7D), up to day $t-l$ included (e.g. accumulated rainfall between day $t-l-6$ and $t-l$); $l_0 = 1$ and $L = 10$ are the minimum and maximum lagged effects of AR7D on cholera incidence. The bi-dimensional rainfall-lag-response function $f.w(x_{i,t-l}, l)$ is composed of two marginal functions: the standard rainfall-response function $f(x)$, and the additional lag-response function $w(l)$. They represent the temporal change in risk after a specific exposure (i.e. AR7D) including the distribution of immediate and delayed effects that cumulate across the lag period.

Both marginal functions were modeled by penalized splines (P-splines) with 10 degrees of freedom. Indeed, the idea underlying penalized DLNM is to form a richly parameterized cross-basis and then to apply penalties to smooth the rainfall-lag-response surface. For ease of interpretation, we calculated the overall rainfall-response

association by cumulating the risk during the 10-day lag period and centering it to a reference value corresponding to the absence of rain (e.g. AR7D = 0).

We fit our model using a quasi-Poisson Generalized Additive Mixed Modelling (GAMM) framework.[12,13] The quasi-Poisson link function allow for potential overdispersion in the cases counts. We make use of the built-in model selection procedures for GAMMs[14] to infer the shape of the P-splines of the rainfall-lag-response structure. Following previous works[10,15], we used restricted maximum likelihood (REML) for estimation of the smoothing parameters. The REML was maximised using the reliable and computationally efficient routines *bam* implemented in the R package *mgcv*.[12,13] The cross-basis and associated penalty matrix (we used the default one) of the DLNM are defined using the R package *dlnm*.[16]

In the main paper we present the results of the regression in terms of relative risks (i.e. the ratio of cholera risk for individuals, cumulated over 10 days after a given AR7D exposure, to the risk when unexposed). We note however that, by construction, our model coefficients are on the same scale as the logged instantaneous reproduction number. One can therefore interpret the relative risks as fold changes (i.e. increase/decrease) of $R_{i,t}$. This is convenient to quantify and compare the contribution of rainfall and other factors to the overall $R_{i,t}$. For instance, the fold change in $R_{i,t}$ attributable to rainfall during the past week, when compared to a week with no rain, is given by $\Delta_{\text{rain}} R_{i,t} = \exp\left(s(x_{i,t-l_0}, \ldots, x_{i,t-L}) - s(0, \ldots, 0)\right)$. Similarly, the fold change in $R_{i,t}$ attributable to Ramadan's specific effects, when compared to the non-Ramadan period, is simply $\Delta_{\text{ram}} R_{i,t} = \exp(\beta_{\text{ram}} + \beta_{\text{ram},i})$.

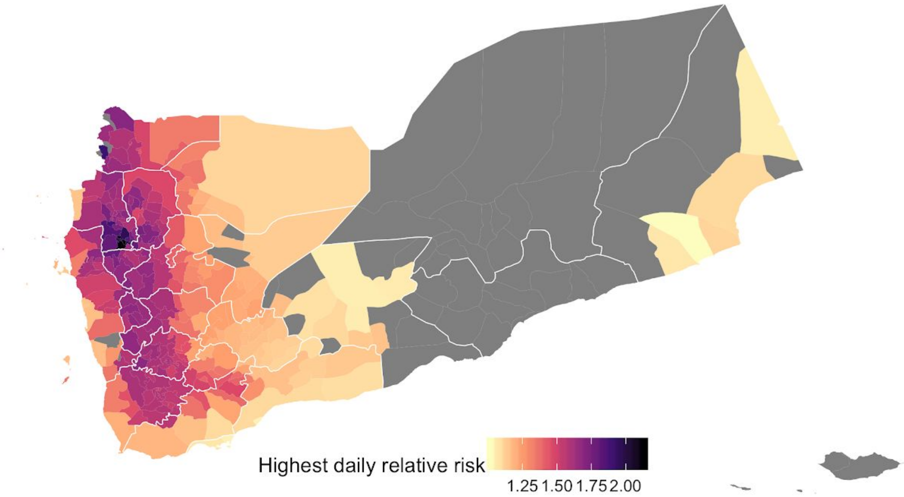

Figure S7: Highest daily relative risk attributable to the accumulated rainfall during the previous 7 days. For each district, the baseline risk corresponds to a typical day following a week with no rain.

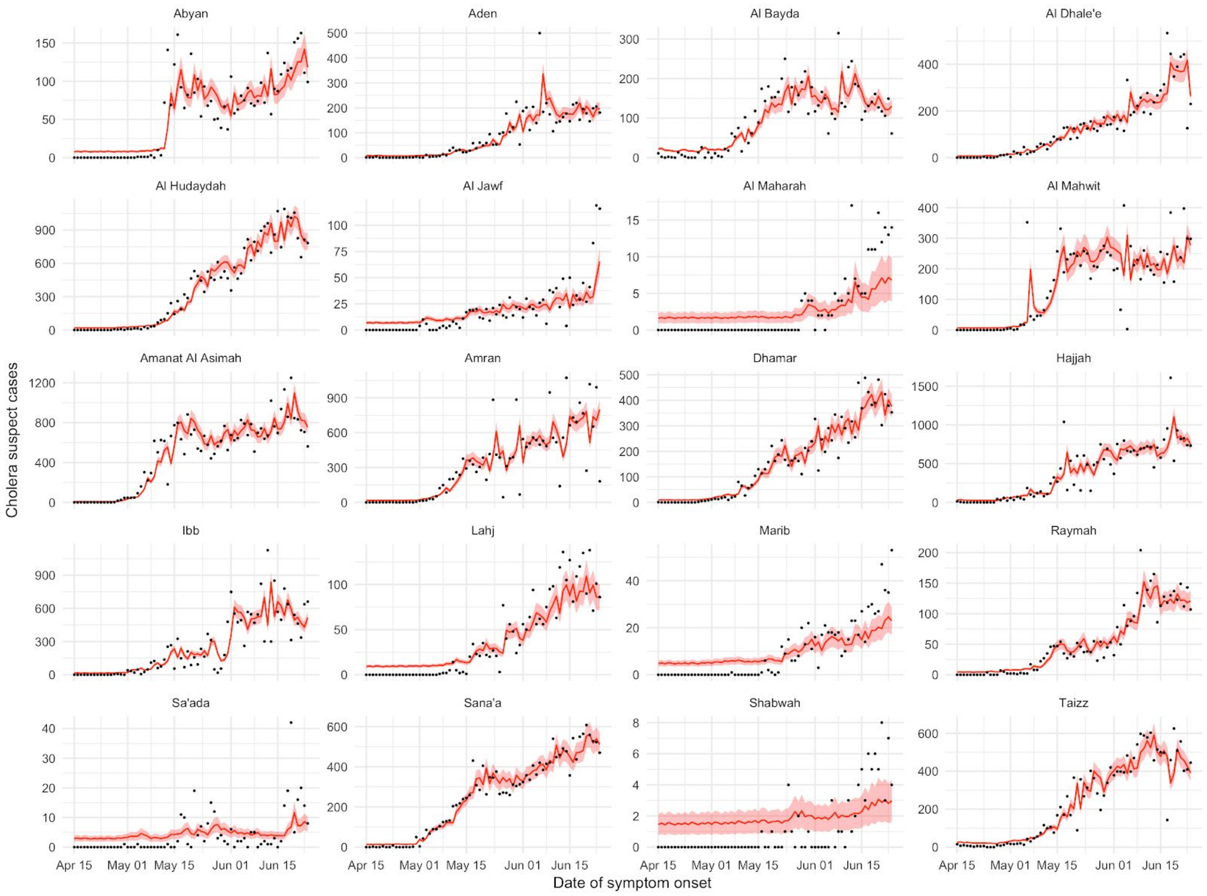

Figure S8: Model fit for each Governorate, computed by aggregating district levels fits. Daily number of cases are represented by black dots, median fit is shown as a red solid line and 95% confidence intervals as red shaded envelopes. Note the free y-axis for each Governorate.

We explored how alternative assumptions regarding the rainfall accumulation and lag periods affected the estimated rainfall-cholera relationship. First, we tested a model with a reduced duration of accumulated rainfall of 4 days, compared to 7 days used in the main analyses (model M2). Second we tested a model with a longer lag effect of 21 days, compared to 10 days used in the main analyses (model M3).

We found similar profiles of relative risk over the following 10 days for the main model and model M2, the slight shift of the peak value arising from the shorter period of accumulated rainfall in M2 (Figure S9 A-B). In model M3 we found the relative risk over 21 days was greater than over 10 days for lower value of accumulated rainfall. This suggests that low quantity of rainfall could have lagged-effect beyond 10 days. All 3 models indicates that the relative increase of the cholera risk due to rainfall did not reach values above 1.5 during the increasing phase of the second epidemic wave.

Comparing the mean daily relative risk per district, we found that all 3 models showed qualitatively similar distribution across the country (Figure S9 C). However, model M3 led to slightly higher estimates than the other two models.

All 3 models having similar values for R^2 and proportion of deviance explained, we conclude that the positive association between rainfall and cholera incidence is robust to changes in model specifications.

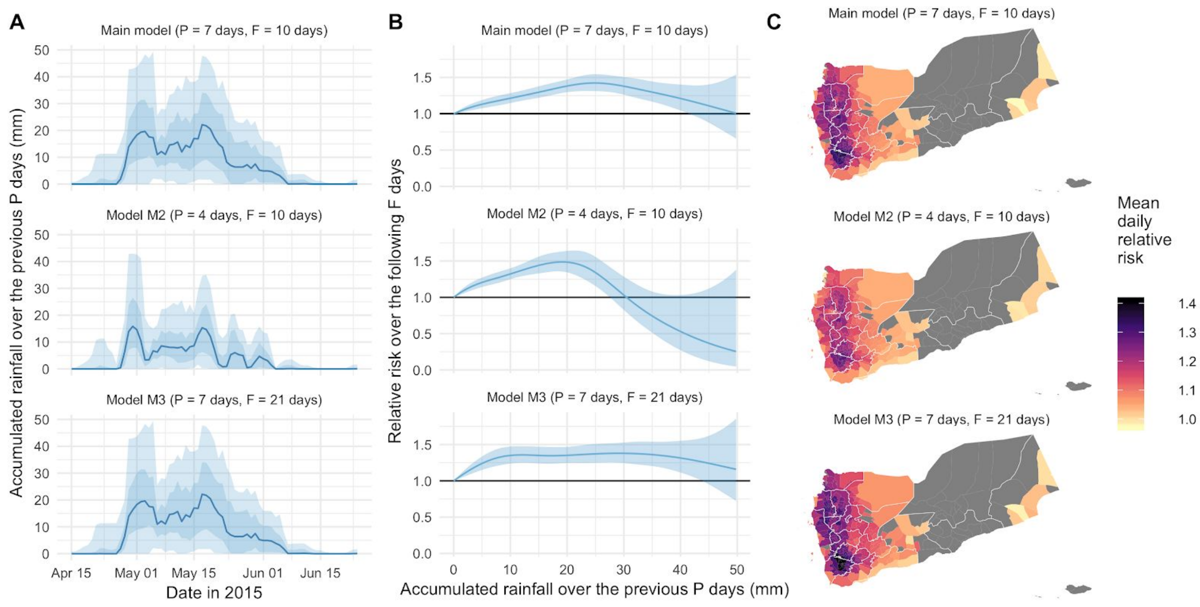

Figure S9: Comparison between the main model and 2 alternative models in their association between rainfall and cholera incidence. **A**: Accumulated rainfall over the 7 (main model and model M3) or 4 (model M2) previous days (AR7D) in millimeters. Solid lines represent the day-wise median over all districts. Dark and light shaded envelopes represent the interquartile range and 95% quantile intervals (centered on the median), respectively. **B**: Relative risk (the ratio of cholera risk for individuals, cumulated over 10 days after a given rainfall exposure, to the risk when unexposed). **C**: Mean daily relative risk attributable to rainfall during the rainy season (districts with no cases reported are in grey). For each district, the baseline risk corresponds to a typical day following a week with no rain.

Spatial heterogeneity in the risk of cholera during the Ramadan period

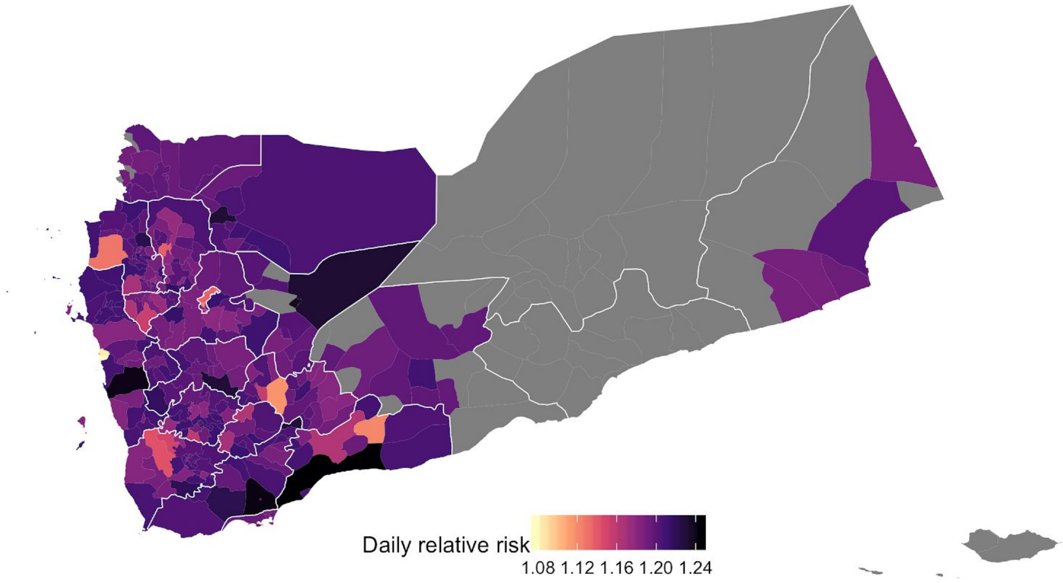

Figure S10: Daily incidence risk-ratio by district attributable to Ramadan-specific effects, in comparison to the month preceding Ramadan. Districts with no cases reported are in grey. The spatial heterogeneity of the relative risk attributable to Ramadan's specific effects was statistically significant in the model.

Rainfall data and interpretation of the model results

CHIRPS incorporates 0.05° resolution satellite imagery with in-situ station data to create gridded rainfall time series. However, as shown in the figure below, since the beginning of the war in 2015 there is no functional ground weather stations in Yemen to calibrate rainfall estimates from satellite imagery.

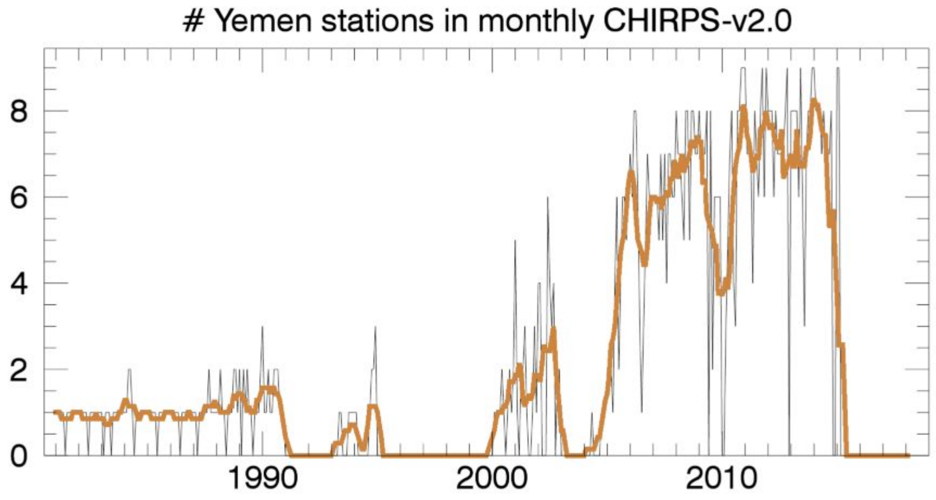

Figure downloaded from CHIRPS website on 5 April 2018.
ftp://ftp.chg.ucsb.edu/pub/org/chg/products/CHIRPS-2.0/diagnostics/stations-perMonth-byCountry/pngs/Yemen.096.station.count.CHIRPS-v2.0.png

More generally, rainfall estimates derived from satellite data provide aerial averages that suffer from bias due to complex terrain which often underestimates precipitation events. Such a bias do not invalidate our findings that increasing amount of rainfall led to an increasing risk of getting cholera (Figure 4), which is probably due to the increasing risk of flooding and use of contaminated water sources. In addition, we found that above a certain amount of rainfall this risk appears to plateau or even decrease (due to the scarcity of days with high rainfall the confidence intervals are huge). This could reflect wash-out effects due to dilution of bacteria concentration in the environment.[17] With the CHIRPS estimation, this effect is reached at 25 mm of estimated rainfall over 7 days, but this would need to be confirmed by ground weather station data.

Vulnerability factors

In June 2017, the WHO country office assessed the vulnerability of the 333 districts of Yemen to cholera by combining 46 indicators from different sources. The indicators were grouped into 8 categories: hazards, impact on populations exposed to the civil war, health-system capacity, morbidity, nutrition, food security, water, sanitation and hygiene (WASH) measures, and social determinants. Sources were UN, UNICEF, the Task Force on Population Movements, the Yemen Health Authorities, the Health Resources and Services Availability Monitoring, the Food Security and Nutrition Assessment, Nutrition and WASH clusters and the last Demographic Health-Survey, from 2013. Most indicators were available at district level except 18 that were only available at governorate level.

We used a quasi-Poisson generalized linear model to estimate the potential associations between the vulnerability indicators and the attack-rates in the 305 districts that reported at least one case during the second epidemic wave. We included several rainfall related indicators for the period April - June 2017 (total rainfall, maximum daily rainfall, total number of rainy days) as well as which side (Houtis or Coalition) was controlling the district in June 2017. We added an offset equal to the logged population size of the districts. Because of the

large number of covariates (more than 50), we followed a two-step approach. First, we ran univariate analyses on each covariate and kept only those with a *p*-value below 0.2 to run a multivariate analysis.

We found that severe acute malnutrition (SAM) was significantly associated with higher attack-rates, with an IRR of 1.34 ([1.11 - 1.63], p = 0.003) per 10 percentage points increase of the percentage of the population under 5y treated for SAM. Two additional variables were positively associated with higher attack-rates: the maximum accumulated rains over consecutive days between April and June 2017 (IRR = 1.08 [1.01 - 1.16], p = 0.025] per 10 millimeters increase of rain) and under 5 years mortality (IRR = 1.94 [1.12 - 3.37], p = 0.019] per 10 units increase of the infant mortality rate).

Districts with active transmission

We used results from 3119 RDTs performed during the last 3 weeks of available data (from 20 February 2018 to 12 March 2018) in order to infer in which districts cholera transmission was likely to be still ongoing at that time. Only 2 culture results were already available for this period, while 28 were awaiting results. This likely reflects the delays from sending of the samples to the national lab to entering the results in the national database. Following previous empirical estimates [18], we assumed that RDT was 92.2% sensitive and 70.6% specific, whereas culture was 66% sensitive and 100% specific. Then we used the R package *RSurveillance*[19] to estimate the true prevalence of cholera and confidence limits for each district and each test (given sample size and sensitivity/specificity of the test). District transmissions were then classified into 4 ordered classes: stopped (prevalence = 0 and upper 95% confidence bound = 0), possibly stopped (prevalence = 0 and upper 95% confidence bound > 0), possibly ongoing (prevalence > 0 and lower 95% confidence bound = 0) and ongoing (prevalence > 0 and lower 95% confidence bound > 0). Final district classification was defined as the highest classification of RDT and culture classifications. In addition, 164 of 333 (49%) districts did not report cholera suspected cases during the considered period, whereas 36 (11%) districts did not perform any RDT or culture for their suspected cases.

RDT and culture confirmation reveal that, as of 12 March 2018, cholera transmission is still active in 11 districts located in the Governorates of Ibb, Amanat Al Asimah, Al Hudaydah, Al Jawf, Al Bayda, Sana'a and Al Mahwit. The Governorates of Ibb, Amanat Al Asimah and Al Hudaydah are the most populated in Yemen, with more than 3 million inhabitants each, mostly located in cities. In addition, there are 46 districts with potential active transmission that would require additional testing for confirmation.

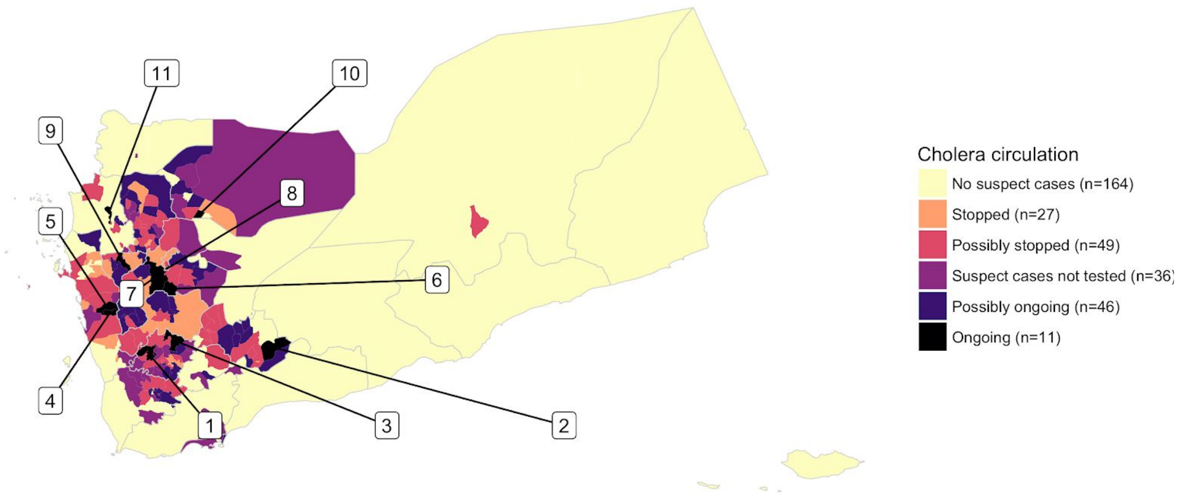

Figure S11: Cholera circulation status for each district based on RDT and culture results performed during the last 3 weeks of available data (20 February 2018 - 12 March 2018). The eleven districts (and their governorate) with ongoing transmission are labelled as follows: 1: Hazm Al Udayn (Ibb), 2: As Sawma Ah (Al Bayda), 3:

Yarim (Ibb), 4: Al Mansuriyah (Al Hudaydah), 5: As Sukhnah (Al Hudaydah), 6: Bilad Ar Rus (Sana'a), 7: Bani Matar (Sana'a), 8: As Sabain (Amanat Al Asimah), 9: Al Mahwait (Al Mahwit), 10: Al Ghayl (Al Jawf), 11: Khayran Al Muharraq (Hajjah).

Assessing the risk of a third epidemic wave in Yemen

One key component of the risk of cholera resurgence is the proportion of the population that remains susceptible to cholera. While precisely estimating this is likely an impossible task with our current understanding of immunity to cholera, we sought to make simple crude estimates using the following conservative assumptions (i.e., those that would lead towards lower estimates of the epidemic risk):

- *Reproductive number*: The basic reproductive number (R_0) is directly related to the herd-immunity threshold (HIT), that is the minimum proportion of the population that must be immune to avoid an epidemic to take-off. Under the assumption of random mixing of the population, we have the following relation: $\mathrm{HIT} = 1 - 1/R_0$ with higher R_0 leading to the need for a higher proportion of immune people to stop transmission. Based on estimates obtained for the second epidemic wave (Figure 3 of the main paper), we assessed 3 different values of R_0 for the third epidemic wave: R_0 equals to 1.2, 1.6 and 2, corresponding to a HIT of 17%, 38% and 50%, respectively.
- *Under-reporting*: We assumed that only a fraction of cholera infections were reported in the surveillance system across the entire epidemic, which includes both cases that did not seek care and asymptomatic infections (assumed to elicit the same protection as symptomatic infections). The lower the under-reporting, the higher the level of immunity in the population and the lower the risk of a third epidemic wave. Here, we assessed reporting rates values of 50% and 20%.
- *Duration of Protection:* While the duration of natural protection is likely on the order of 5-10 years, these rough calculations assume that protection at least lasts 1.5 years (Oct-2016 through April-2018). Put another way, individuals infected during the first two waves were assumed to be fully protected for the third wave.
- *V. cholerae Strain:* We assume that any third wave will be caused by the same *V cholerae* O1 El Tor Ogawa strain. Given that Ogawa serotypes have imperfect cross-protection with Inaba serotypes,[20] a switch in serotype, which can occur within an epidemic,[21] would mean that we overestimate the proportion of the population immune.
- *Place of resurgence*: We assume that cholera will resurge in districts previously affected during the first two epidemic waves. That is 305/333 Yemen districts, hence we make the conservative assumptions that the risk is null in the other 28 districts, totalling a population of 927,252.

Under the most conservative combination of a reporting rate of 20% and an R_0 of 1.2, we found that 54% of the districts were below the HIT, totalling a population at risk of more than 13.8 million, which represents almost 50% of the population in the 305 districts previously affected. Assuming higher R_0 values (1.6 or 2), comparable to those estimated at the outset of the second wave, we found that more than 80% of the districts and more than 75% of the population remained at risk in case of cholera re-introduction. Increasing the reporting rate from 20% to 50% led to nearly all districts becoming at risk of a third epidemic wave (Table S1 and Figure S12). In conclusion, despite the huge epidemic in 2016-2017, most people have not been affected and most districts can likely sustain transmission if cholera is re-introduced and transmission returns to similar levels as during the 2017 spring rainy season.

Table S1: Population at risk of a third epidemic wave in the 305 districts that were affected during the first two waves (September 28, 2016 to March 12, 2018).

R_0 [1]	HIT [3]	Districts at risk		Individuals at risk	
		n	% [4]	n	% [4]
**Reporting rate[4] = 20%**					
1.2	17%	166	54.4%	13,889,396	48%
1.6	38%	256	83.9%	21,648,017	75%
2	50%	288	94.4%	23,073,270	80%
**Reporting rate[4] = 50%**					
1.2	17%	265	86.9%	24,519,299	85%
1.6	38%	304	99.7%	26,794,254	92%
2	50%	305	100%	26,804,571	92%

[1]Assumed value of the basic reproduction number at the beginning of the 3rd epidemic wave; [2]Herd-Immunity Threshold; [3]Calculated among the population of the 305 districts affected by the 1st and 2nd epidemic waves; [4]Assumed proportion of cholera infections reported in the surveillance system during the 1st and 2nd epidemic waves

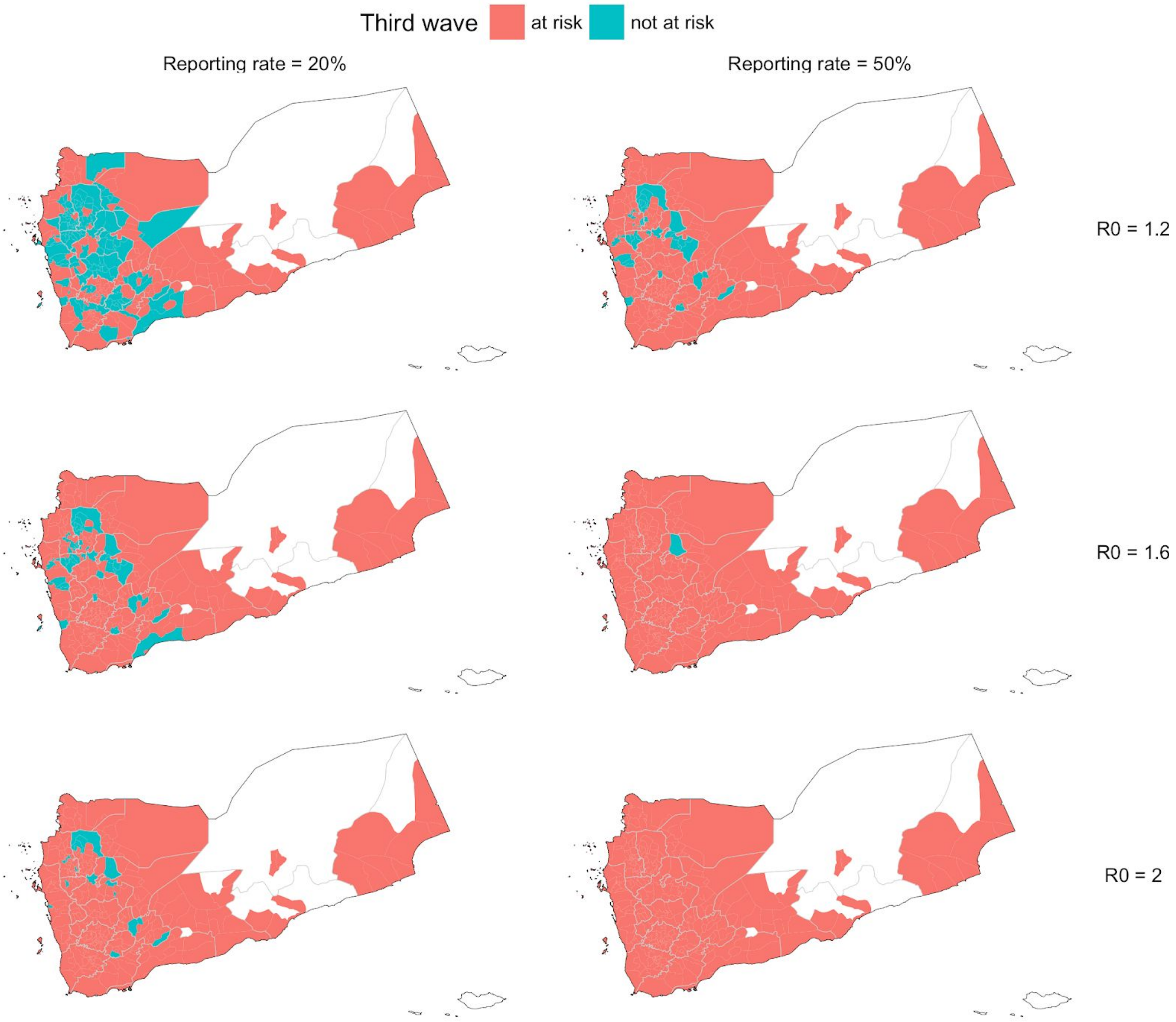

Figure S12: Districts at risk of a third epidemic wave under different assumptions for the reporting rate during the first two waves (September 28, 2016 to March 12, 2018) and the basic reproduction number at the outset of the third wave. We consider only the 305/333 districts previously affected during the first two waves (areas in white represent those not previously affected).

References

1 UN World Population Prospects 2017. UN World Population Prospects 2017. https://esa.un.org/unpd/wpp/ (accessed Jan 2018).

2 UN Office for the Coordination of Humanitarian Affairs, UN Country Team in Yemen. Humanitarian Needs Overview. UNOCHA, 2017 https://reliefweb.int/sites/reliefweb.int/files/resources/yemen_humanitarian_needs_overview_hno_2018_20171204_0.pdf.

3 Cori A, Ferguson NM, Fraser C, Cauchemez S. A new framework and software to estimate time-varying reproduction numbers during epidemics. *Am J Epidemiol* 2013; **178**: 1505–12.

4 Fraser C. Estimating Individual and Household Reproduction Numbers in an Emerging Epidemic. *PLoS One* 2007; **2**: e758.

5 Weil AA, Khan AI, Chowdhury F, *et al.* Clinical outcomes in household contacts of patients with cholera in Bangladesh. *Clin Infect Dis* 2009; **49**: 1473–9.

6 Azman AS, Rumunu J, Abubakar A, *et al.* Population-Level Effect of Cholera Vaccine on Displaced Populations, South Sudan, 2014. *Emerg Infect Dis* 2016; **22**: 1067–70.

7 Imai C, Armstrong B, Chalabi Z, Mangtani P, Hashizume M. Time series regression model for infectious disease and weather. *Environ Res* 2015; **142**: 319–27.

8 Koelle K, Pascual M. Disentangling extrinsic from intrinsic factors in disease dynamics: a nonlinear time series approach with an application to cholera. *Am Nat* 2004; **163**: 901–13.

9 Koelle K, Rodó X, Pascual M, Yunus M, Mostafa G. Refractory periods and climate forcing in cholera dynamics. *Nature* 2005; **436**: 696–700.

10 Gasparrini A, Scheipl F, Armstrong B, Kenward MG. A penalized framework for distributed lag non-linear models. *Biometrics* 2017; **73**: 938–48.

11 Gasparrini A, Armstrong B, Kenward MG. Distributed lag non-linear models. *Stat Med* 2010; **29**: 2224–34.

12 Wood SN. Generalized Additive Models: An Introduction with R, Second Edition. CRC Press, 2017.

13 R Core Team. R: A Language and Environment for Statistical Computing. R Foundation for Statistical Computing, 2018 https://www.R-project.org.

14 Wood SN. Low-rank scale-invariant tensor product smooths for generalized additive mixed models. *Biometrics* 2006; **62**: 1025–36.

15 Wood SN. Fast stable restricted maximum likelihood and marginal likelihood estimation of semiparametric generalized linear models. *J R Stat Soc Series B Stat Methodol* 2010; **73**: 3–36.

16 Gasparrini A. Distributed Lag Linear and Non-Linear Models inR: The Packagedlnm. *J Stat Softw* 2011; **43**. DOI:10.18637/jss.v043.i08.

17 Ruiz-Moreno D, Pascual M, Bouma M, Dobson A, Cash B. Cholera Seasonality in Madras (1901–1940): Dual Role for Rainfall in Endemic and Epidemic Regions. *Ecohealth* 2007; **4**: 52–62.

18 Page A-L, Alberti KP, Mondonge V, Rauzier J, Quilici M-L, Guerin PJ. Evaluation of a Rapid Test for the Diagnosis of Cholera in the Absence of a Gold Standard. *PLoS One* 2012; **7**: e37360.

19 Sergeant E. RSurveillance: Design and Analysis of Disease Surveillance Activities. 2016 https://CRAN.R-project.org/package=RSurveillance.

20 Ali M, Emch M, Park JK, Yunus M, Clemens J. Natural Cholera Infection–Derived Immunity in an Endemic Setting. *J Infect Dis* 2011; **204**: 912–8.

21 Karlsson SL, Thomson N, Mutreja A, *et al.* Retrospective Analysis of Serotype Switching of Vibrio cholerae O1 in a Cholera Endemic Region Shows It Is a Non-random Process. *PLoS Negl Trop Dis* 2016; **10**: e0005044.
